# Supplementary material for: Thermodynamics and kinetics guided probe design for uniformly sensitive and specific DNA hybridization without optimization
Source: Nat Commun. 2019 Oct 14;10:4675. doi: 10.1038/s41467-019-12593-9 (PMC6791858; doi:10.1038/s41467-019-12593-9)
Supplement: Supplementary file 1 — Supplementary information [file 41467_2019_12593_MOESM1_ESM.pdf]

Thermodynamics and kinetics guided probe design for uniformly sensitive and specific DNA hybridization without optimization

Chen et al.

Supplementary Methods.

Materials. Oligonucleotides used were purchased from Sangon Biotech (Shanghai); The detailed sequences are listed in Supplementary Table 9 to Supplementary Table 14. Oligonucleotides were checked by Sangon Biotech for purity by capillary electrophoresis and by electrospray ionization mass spectrometry for identity. All labeled strands (F and Q) were HPLC purified by Sangon Biotech; Oligonucleotides were ordered in dry format, and suspended in Tris-EDTA buffer.

Q5 High-Fidelity DNA polymerase (Q5 polymerase), Q5 reaction buffer, Lambda exonuclease (λ exo), Lambda Exonuclease buffer (67 mM Glycine-KOH, 2.5 mM $MgCl_2$, 50 μg ml^{-1} BSA, pH 9.4 @ 25℃), ThermoPol Reaction Buffer (20 mM Tris–HCl, 10 mM KCl, 10 mM $(NH_4)_2SO_4$, 2 mM $MgSO_4$ and 0.1% Triton X-100, pH 8.8 @ 25℃), dNTPs and DNA Purple Loading Dye were purchased from New England Biolabs (MA, USA). Low-MW DNA marker was purchased from Sangon Biotech Corporation (Shanghai, China). GelRed Dye was purchased from Biotumn (CA, USA). DNase/RNase free deionized water was purchased from Tiangen Biotech Corporation (Beijing, China). Genomic DNA extracted from blood sample of a healthy volunteer was used as WT DNA control. The blood and tissue samples were obtained from cancer patients in Union Hospital, Huazhong University of Science and Technology.

Theoretical modelling. The thermodynamic parameters (free energy change) were obtained through NUPACK. Symbolic computation for deriving the analytic expressions of sensitivity and specificity were performed on Maple 2019. Accurate solutions of equilibrium concentrations with all parameters known and introduced were also calculated through Maple 2019. The number of output solutions could be tuned by setting the interval width.

Reaction Setup for probe/blocker composition system. To a PCR tube, the L-strand and S-strand were mixed and annealed to prepare the probe solution. To make sure that the quencher labelled L-strand was fully bound to fluorophore labelled S-strand, we set the amount of S-strand 25% higher than that of L-strand.

To plate strip, appropriate amount of Probe, Blocker, WT and MT solution were added. Then the plate strip were immediately put into a microplate reader (Biotek) for fluorescence measurement. The amount of Probe, Blocker, MT, and WT added in different competitive composition systems are listed in Supplementary Table 1.

Supplementary Table 1. Reaction setups for investigating the influence of $-\Delta G_{BW}$ and $[Blocker]_0$ on the performance.

System	Experiment	Concentrations of reactants (nM)				
		Probe	Blocker	MT	WT	Buffer
Strand displacement probe/standard blocker system	Changing the $-\Delta G_{BW}$	1000	2400	250	250	1 x
	Changing the $[B]_0$	1000	300 | 600 | 1200 | 2400 | 4800 | 9600 | 19200	250	250	1 x
Dissociative 4-Way SELECT system	Changing the $-\Delta G_{BW}$	1000	2000	250	250	1 x
	Changing the $[B]_0$	1000	250 | 500 | 1000 | 2000 | 4000 | 8000	250	250	1 x
Non-dissociative 4-Way SELECT system	Changing the $-\Delta G_{BW}$	1000	4000	500	500	1 x
	Changing the $[B]_0$	1000	500 | 1000 | 2000 | 4000 | 8000 | 16000	500	500	1 x

Supplementary Table 2. Reaction setups for measurement of IF.

Experiment	Concentrations of reactants (nM)					
	Probe	Blocker	MT	WT	NT	Buffer
F_a	20	8000	10	990	-	1 x
F_b	20	8000	-	1000	-	1 x
F_c	20	8000	-	-	1000	1 x

Supplementary Table 3. Reaction setups for identification of SNVs.

Experiment	Concentrations of reactants (nM)						
	Probe	Shared-FAM	Shared-BHQ	Blocker	MT	WT	Buffer
Multiplexed identification of SNVs	1000	1000	1000	2000	250	250	1 x

Fluorescence Data Analysis. Data acquired by the Gen5™ software was exported to a EXCEL file, which was subsequently imported, analyzed, and plotted using MATLAB scripts and ORIGIN. Time was linearly adjusted so that t = 0 corresponds to the first data point acquired after the plate strips were put into the machine after addition of reagents. No additional adjustments are made to the raw fluorescence kinetics plots in the Supplementary Figure 5 to Supplementary Figure 14.

Post-PCR genotyping and detection of low-abundance mutations. We first measured the concentrations of the human genomic DNA samples NA18537 (SMAD7-T homozygote) by Nanodrop 2000c spectrophotometer (Thermo Fisher). The sample was then diluted to a concentration of 50 ng μl^{-1}, and 1 µL of the diluted sample was added into a PCR tube to achieve a final concentration of 2.5 ng μl^{-1} (20 µL). After PCR, Exonuclease 1 (NEB) and Lambda exo (NEB) were added to degrade the double stranded PCR products and obtain single stranded DNA for further analysis. The detailed reactants for PCR were listed in Supplementary Table 4, and the thermal profiles were illustrated in Supplementary Table 5. The single stranded DNA were collected by a DNA purification kit (TIANquick Midi Purification Kit). After purification, the targeted single stranded DNA were treated with the probe/blocker composition system for fluorescence measurement.

Supplementary Table 4. Formulation of PCR reactions.

Reactants	Final Concentration
10 x PCR buffer	1x
dNTP (each)	200 µM
Forward Primer	2 µM
Reverse Primer	2 µM
Taq Polymerase	0.05 Unit/µL
Mixed gDNA	2.5 ng/µl
Nuclease-free Water	Fill to 20 µL

Supplementary Table 5. Thermal profile for preparation of single stranded DNA analyte.

	Step	Temperature	Duration
PCR amplification (repeat step 2-4 for 40 cycles)	1. Initial Denaturation	95 ℃	2min
	2. Denaturation	95 ℃	30s
	3. Annealing	95 ℃	30s
	4. Extension	95 ℃	30s
	5. Final Extension	72 ℃	5 min
Prepare single-stranded PCR products for further analysis	6. Exo I digestion	37 ℃	15min
	7. Inactivation of Exo I	85 ℃	15min
	8. λ exo digestion	37 ℃	15min
	9. Inactivation of λ exo	75 ℃	15min

Supplementary Note 1.

Thermodynamic model and analysis for standard probe/standard blocker system.

The reactions in the MT solution were,

$$\mathrm{MT}+\mathrm{B} \underset{}{\overset{K_{\mathrm{BM}}}{\rightleftharpoons}} \mathrm{BMT}$$

$$\mathrm{MT}+\mathrm{P} \underset{}{\overset{K_{\mathrm{PM}}}{\rightleftharpoons}} \mathrm{PMT}$$

The reactions in the WT solution were,

$$\mathrm{WT}+\mathrm{B} \underset{}{\overset{K_{\mathrm{BW}}}{\rightleftharpoons}} \mathrm{BWT}$$

$$\mathrm{WT}+\mathrm{P} \underset{}{\overset{K_{\mathrm{PW}}}{\rightleftharpoons}} \mathrm{PWT}$$

According to the mass-action equilibria,

$$\frac{[\mathrm{BMT}]}{[\mathrm{MT}][\mathrm{B}]}=K_{\mathrm{BM}} \quad (1) \qquad \frac{[\mathrm{BWT}]}{[\mathrm{WT}][\mathrm{B}]}=K_{\mathrm{BW}} \quad (2)$$

$$\frac{[\mathrm{PMT}]}{[\mathrm{MT}][\mathrm{P}]}=K_{\mathrm{PM}} \quad (3) \qquad \frac{[\mathrm{PWT}]}{[\mathrm{WT}][\mathrm{P}]}=K_{\mathrm{PW}} \quad (4)$$

$$[\mathrm{MT}]+[\mathrm{PMT}]+[\mathrm{BMT}]=c_0 \quad (5) \qquad [\mathrm{WT}]+[\mathrm{PWT}]+[\mathrm{BWT}]=c_0 \quad (6)$$

$$[\mathrm{PMT}]+[\mathrm{P}]=[\mathrm{P}]_0 \quad (7) \qquad [\mathrm{PWT}]+[\mathrm{P}]=[\mathrm{P}]_0 \quad (8)$$

$$[\mathrm{BMT}]+[\mathrm{B}]=[\mathrm{B}]_0 \quad (9) \qquad [\mathrm{BWT}]+[\mathrm{B}]=[\mathrm{B}]_0 \quad (10)$$

As was described in the main article, we defined sensitivity and specificity as,

$$\text{Sensitivity}=\frac{[\mathrm{PMT}]}{c_0} \quad (11)$$

$$\text{Specificity}=\frac{[\mathrm{PMT}]}{[\mathrm{PWT}]} \quad (12)$$

Combining equations above, we could obtain the expression of $[\mathrm{PMT}]$ and $[\mathrm{PWT}]$.

$$[\mathrm{PMT}]=\frac{[\mathrm{P}]K_{\mathrm{PM}}c_0}{[\mathrm{B}]K_{\mathrm{BM}}+[\mathrm{P}]K_{\mathrm{PM}}+1} \quad (13)$$

$$[\mathrm{PWT}]=\frac{[\mathrm{P}]K_{\mathrm{PW}}c_0}{[\mathrm{B}]K_{\mathrm{BW}}+[\mathrm{P}]K_{\mathrm{PW}}+1} \quad (14)$$

Derivation of $[\mathrm{PMT}]$,

$$\frac{\partial[\mathrm{PMT}]}{\partial[\mathrm{B}]}=\frac{K_{\mathrm{BM}}K_{\mathrm{PM}}c_0\left(-[\mathrm{P}]_0+[\mathrm{PMT}]\right)}{\left(\begin{array}{l}[\mathrm{B}]^2K_{\mathrm{BM}}{}^2+2[\mathrm{B}]K_{\mathrm{BM}}+[\mathrm{B}]K_{\mathrm{BM}}K_{\mathrm{PM}}c_0+2[\mathrm{B}]K_{\mathrm{BM}}K_{\mathrm{PM}}\left([\mathrm{P}]_0-[\mathrm{PMT}]\right)+\\ \left(K_{\mathrm{PM}}[\mathrm{P}]_0-K_{\mathrm{PM}}[\mathrm{PMT}]\right)^2+2K_{\mathrm{PM}}\left([\mathrm{P}]_0-[\mathrm{PMT}]\right)+K_{\mathrm{PM}}c_0+1\end{array}\right)} \quad (15)$$

Since,

$$[\mathrm{PMT}]-[\mathrm{P}]_0<0 \quad (16)$$

We have,

$$\frac{\partial[\mathrm{PMT}]}{\partial[\mathrm{B}]}<0. \quad (17)$$

Therefore, the function of $[\mathrm{PMT}]$ and Sensitivity over $[\mathrm{B}]$ is monotonically decreasing.

We also could use proof by contradiction to demonstrate that $[\mathrm{PMT}]$ and sensitivity is monotonically decreasing

with K_{BM}:

Consider an increase in K_{BM}, we want to prove that $[\mathrm{PMT}]$ necessarily decreases.

For contradiction, assume not,

$$[\mathrm{PMT}] \geq [\mathrm{PMT}_{\mathrm{old}}] \tag{18}$$

Since,

$$[\mathrm{PMT}] + [\mathrm{P}] = [\mathrm{P}]_0 \equiv \mathrm{constant} \tag{19}$$

Thus,

$$[\mathrm{P}] \leq [\mathrm{P}_{\mathrm{old}}] \tag{20}$$

According to the mass-action equilibria for PMT formation,

$$\frac{[\mathrm{PMT}]}{[\mathrm{MT}][\mathrm{P}]} = K_{\mathrm{PM}} \tag{21}$$

Combine the above four equations,

$$[\mathrm{MT}] \geq [\mathrm{MT}_{\mathrm{old}}] \tag{22}$$

Since,

$$[\mathrm{MT}] + [\mathrm{PMT}] + [\mathrm{BMT}] = c_0 \tag{23}$$

Then,

$$[\mathrm{BMT}] \leq [\mathrm{BMT}_{\mathrm{old}}] \tag{24}$$

Similarly,

$$[\mathrm{BMT}] + [\mathrm{B}] = [\mathrm{B}]_0 \equiv \mathrm{constant} \tag{25}$$

Thus,

$$[\mathrm{B}] \geq [\mathrm{B}_{\mathrm{old}}] \tag{26}$$

However, $[\mathrm{MT}] \geq [\mathrm{MT}_{\mathrm{old}}]$, $[\mathrm{B}] \geq [\mathrm{B}_{\mathrm{old}}]$ and $[\mathrm{BMT}] \leq [\mathrm{BMT}_{\mathrm{old}}]$ lead to

$$K_{\mathrm{BM}} = \frac{[\mathrm{BMT}]}{[\mathrm{MT}][\mathrm{B}]} \leq \frac{[\mathrm{BMT}_{\mathrm{old}}]}{[\mathrm{MT}_{\mathrm{old}}][\mathrm{B}_{\mathrm{old}}]} = K_{\mathrm{BM}_{\mathrm{old}}} \tag{27}$$

The above equation is contradictory to the initial condition` of an increase in K_{BM}. Conclusively, we have proved that the sensitivity is monotonically decreasing over $[\mathrm{B}]$ and K_{BM}.

However, for specificity, we have to adopt the following approximations:

$$[\mathrm{B}] = [\mathrm{B}]_0 - c_0 \tag{28}$$

$$[\mathrm{P}] = [\mathrm{P}]_0 \tag{29}$$

Then, we could obtain much simpler analytic expressions of sensitivity and specificity over $[\mathrm{B}]_0$ and K_{BM},

$$\mathrm{Sensitivity} = \frac{[\mathrm{P}]_0 K_{\mathrm{PM}}}{\left([\mathrm{B}]_0 - c_0\right) K_{\mathrm{BM}} + [\mathrm{P}]_0 K_{\mathrm{PM}} + 1} \tag{30}$$

$$\mathrm{Specificity} = \frac{K_{\mathrm{PM}}}{K_{\mathrm{PW}}} \times \frac{\left([\mathrm{B}]_0 - c_0\right) K_{\mathrm{BW}} + [\mathrm{P}]_0 K_{\mathrm{PW}} + 1}{\left([\mathrm{B}]_0 - c_0\right) K_{\mathrm{BM}} + [\mathrm{P}]_0 K_{\mathrm{PM}} + 1} \tag{31}$$

The relations between the involved free energy changes and equilibrium constants were as follows,

$$\Delta G_{\mathrm{PM}} = \Delta G_{\mathrm{PW}} - \Delta\Delta G_{\mathrm{P}} \tag{32}$$

$$\Delta G_{\mathrm{BM}} = \Delta G_{\mathrm{BW}} + \Delta\Delta G_{\mathrm{B}} \tag{33}$$

$$K = \mathrm{e}^{-\frac{\Delta G}{RT}} \tag{34}$$

The analytic expressions of sensitivity and specificity could be further transformed into,

$$\text{Sensitivity} = \frac{[\mathrm{P}]_0 \mathrm{e}^{-\frac{\Delta G_{\mathrm{PM}}}{RT}}}{\mathrm{e}^{-\frac{\Delta G_{\mathrm{BW}} + \Delta\Delta G_{\mathrm{B}}}{RT}} \left([\mathrm{B}]_0 - c_0\right) + [\mathrm{P}]_0 \mathrm{e}^{-\frac{\Delta G_{\mathrm{PM}}}{RT}} + 1} \tag{35}$$

$$\text{Specificity} = \mathrm{e}^{\frac{\Delta\Delta G_{\mathrm{P}}}{RT}} \times \frac{\left([\mathrm{B}]_0 - c_0\right)\mathrm{e}^{-\frac{\Delta G_{\mathrm{BW}}}{RT}} + [\mathrm{P}]_0 \mathrm{e}^{-\frac{\Delta G_{\mathrm{PM}} + \Delta\Delta G_{\mathrm{P}}}{RT}} + 1}{\left([\mathrm{B}]_0 - c_0\right)\mathrm{e}^{-\frac{\Delta G_{\mathrm{BW}} + \Delta\Delta G_{\mathrm{B}}}{RT}} + [\mathrm{P}]_0 \mathrm{e}^{-\frac{\Delta G_{\mathrm{PM}}}{RT}} + 1} \tag{36}$$

Herein, we defined μ as,

$$\mu = [\mathrm{B}]\mathrm{e}^{-\frac{\Delta G_{\mathrm{BW}}}{RT}} = \left([\mathrm{B}]_0 - c_0\right)\mathrm{e}^{-\frac{\Delta G_{\mathrm{BW}}}{RT}} \tag{37}$$

Then,

$$\text{Sensitivity} = \frac{[\mathrm{P}]_0 \mathrm{e}^{-\frac{\Delta G_{\mathrm{PM}}}{RT}}}{\mu \mathrm{e}^{-\frac{\Delta\Delta G_{\mathrm{B}}}{RT}} + [\mathrm{P}]_0 \mathrm{e}^{-\frac{\Delta G_{\mathrm{PM}}}{RT}} + 1} \tag{38}$$

$$\text{Specificity} = \mathrm{e}^{\frac{\Delta\Delta G_{P}}{RT}} \times \frac{\mu + [\mathrm{P}]_0 \mathrm{e}^{-\frac{\Delta G_{\mathrm{PM}} + \Delta\Delta G_{\mathrm{P}}}{RT}} + 1}{\mu \mathrm{e}^{-\frac{\Delta\Delta G_{\mathrm{B}}}{RT}} + [\mathrm{P}]_0 \mathrm{e}^{-\frac{\Delta G_{\mathrm{PM}}}{RT}} + 1} \tag{39}$$

Derivation of sensitivity,

$$\frac{\partial \text{Sensitivity}}{\partial [\mathrm{B}]_0} = \frac{d\text{Sensitivity}}{d\mu} \times \frac{\partial \mu}{\partial [\mathrm{B}]_0} = -\frac{[\mathrm{P}]_0 \mathrm{e}^{-\frac{\Delta G_{\mathrm{PM}} + \Delta\Delta G_{\mathrm{B}}}{RT}}}{\left(\mu \mathrm{e}^{-\frac{\Delta\Delta G_{\mathrm{B}}}{RT}} + [\mathrm{P}]_0 \mathrm{e}^{-\frac{\Delta G_{\mathrm{PM}}}{RT}} + 1\right)^2} \times e^{-\frac{\Delta G_{\mathrm{BW}}}{RT}} < 0 \tag{40}$$

$$\frac{\partial \text{Sensitivity}}{\partial \left(-\Delta G_{\mathrm{BW}}\right)} = \frac{d\text{Sensitivity}}{d\mu} \times \frac{\partial \mu}{\partial \left(-\Delta G_{\mathrm{BW}}\right)} = -\frac{[\mathrm{P}]_0 \mathrm{e}^{-\frac{\Delta G_{\mathrm{PM}} + \Delta\Delta G_{\mathrm{B}}}{RT}}}{\left(\mu \mathrm{e}^{-\frac{\Delta\Delta G_{\mathrm{B}}}{RT}} + [\mathrm{P}]_0 \mathrm{e}^{-\frac{\Delta G_{\mathrm{PM}}}{RT}} + 1\right)^2} \times \frac{\left([\mathrm{B}]_0 - c_0\right)\mathrm{e}^{-\frac{\Delta G_{\mathrm{BW}}}{RT}}}{RT} < 0 \tag{41}$$

Thus, the sensitivity is monotonically decreasing over $[\mathrm{B}]_0$ and $-\Delta G_{\mathrm{BW}}$.
For the derivation of specificity,

$$\frac{\partial \text{Specificity}}{\partial [\mathrm{B}]_0} = \frac{d\text{Specificity}}{d\mu} \times \frac{\partial \mu}{\partial [\mathrm{B}]_0} = \frac{[\mathrm{P}]_0 \mathrm{e}^{-\frac{\Delta G_{\mathrm{PM}} - \Delta\Delta G_{\mathrm{P}}}{RT}} - [\mathrm{P}]_0 \mathrm{e}^{-\frac{\Delta G_{\mathrm{PM}} + \Delta\Delta G_{\mathrm{B}}}{RT}} + \mathrm{e}^{\frac{\Delta\Delta G_{\mathrm{P}}}{RT}} - \mathrm{e}^{\frac{\Delta\Delta G_{\mathrm{P}} - \Delta\Delta G_{\mathrm{B}}}{RT}}}{\left(\mu \mathrm{e}^{-\frac{\Delta\Delta G_{\mathrm{B}}}{RT}} + [\mathrm{P}]_0 \mathrm{e}^{-\frac{\Delta G_{\mathrm{PM}}}{RT}} + 1\right)^2} \times \mathrm{e}^{-\frac{\Delta G_{\mathrm{BW}}}{RT}} \tag{42}$$

$$\frac{\partial \text{Specificity}}{\partial \left(-\Delta G_{\mathrm{BW}}\right)} = \frac{d\text{Specificity}}{d\mu} \times \frac{\partial \mu}{\partial \left(-\Delta G_{\mathrm{BW}}\right)} = \frac{[\mathrm{P}]_0 \mathrm{e}^{-\frac{\Delta G_{\mathrm{PM}} - \Delta\Delta G_{\mathrm{P}}}{RT}} - [\mathrm{P}]_0 \mathrm{e}^{-\frac{\Delta G_{\mathrm{PM}} + \Delta\Delta G_{\mathrm{B}}}{RT}} + \mathrm{e}^{\frac{\Delta\Delta G_{\mathrm{P}}}{RT}} - \mathrm{e}^{\frac{\Delta\Delta G_{\mathrm{P}} - \Delta\Delta G_{\mathrm{B}}}{RT}}}{\left(\mu \mathrm{e}^{-\frac{\Delta\Delta G_{\mathrm{B}}}{RT}} + [\mathrm{P}]_0 \mathrm{e}^{-\frac{\Delta G_{\mathrm{PM}}}{RT}} + 1\right)^2} \times \frac{\left([\mathrm{B}]_0 - c_0\right)\mathrm{e}^{-\frac{\Delta G_{\mathrm{BW}}}{RT}}}{RT} \tag{43}$$

Since $\Delta\Delta G_{\mathrm{P}} > 0, \Delta\Delta G_{\mathrm{B}} > 0$

$$\frac{\partial \text{Specificity}}{\partial [\mathrm{B}]_0} > 0 \tag{44}$$

$$\frac{\partial \text{Specificity}}{\partial\left(-\Delta G_{\text{BW}}\right)} > 0 \tag{45}$$

Thus, the specificity is monotonically increasing over $[\mathrm{B}]_0$ and $-\Delta G_{\text{BW}}$.

$$\lim_{\mu\to 0}\text{Sensitivity}=\frac{\mathrm{e}^{-\frac{\Delta G_{\text{PM}}}{RT}}[\mathrm{P}]_0}{\mathrm{e}^{-\frac{\Delta G_{\text{PM}}}{RT}}[\mathrm{P}]_0+1} \quad \left(\text{Maximum}\right) \tag{46}$$

$$\lim_{\mu\to 0}\text{Specificity} = \mathrm{e}^{\frac{\Delta\Delta G_{\text{P}}}{RT}} \times \frac{[\mathrm{P}]_0\mathrm{e}^{-\frac{\Delta G_{\text{PM}}+\Delta\Delta G_{\text{P}}}{RT}}+1}{[\mathrm{P}]_0\mathrm{e}^{-\frac{\Delta G_{\text{PM}}}{RT}}+1} \quad \left(\text{Minimum}\right) \tag{47}$$

$$\lim_{\mu\to +\infty}\text{Sensitivity} = \frac{[\mathrm{P}]_0\mathrm{e}^{-\frac{\Delta G_{\text{PM}}}{RT}}}{\mu\mathrm{e}^{-\frac{\Delta\Delta G_{\text{B}}}{RT}}+[\mathrm{P}]_0\mathrm{e}^{-\frac{\Delta G_{\text{PM}}}{RT}}+1} \to 0 \quad \left(\text{Minimum}\right) \tag{48}$$

$$\lim_{\mu\to +\infty}\text{Specificity} = \mathrm{e}^{\frac{\Delta\Delta G_{\text{P}}}{RT}} \times \mathrm{e}^{\frac{\Delta\Delta G_{\text{B}}}{RT}} \quad \left(\text{Maximum}\right) \tag{49}$$

To sum up,

$$\text{Sensitivity}([\mathrm{B}]_0, -\Delta G_{\text{BW}}) \in [\frac{\mathrm{e}^{-\frac{\Delta G_{\text{PM}}}{RT}}[\mathrm{P}]_0}{\mathrm{e}^{-\frac{\Delta G_{\text{PM}}}{RT}}[\mathrm{P}]_0+1}, 0) \searrow \tag{50}$$

$$\text{Specificity}([\mathrm{B}]_0, -\Delta G_{\text{BW}}) \in [\mathrm{e}^{\frac{\Delta\Delta G_{\text{P}}}{RT}} \times \frac{[\mathrm{P}]_0\mathrm{e}^{-\frac{\Delta G_{\text{PM}}+\Delta\Delta G_{\text{P}}}{RT}}+1}{[\mathrm{P}]_0\mathrm{e}^{-\frac{\Delta G_{\text{PM}}}{RT}}+1}, e^{\frac{\Delta\Delta G_{\text{P}}}{RT}} \times e^{\frac{\Delta\Delta G_{\text{B}}}{RT}}) \nearrow \tag{51}$$

Supplementary Note 2.

Thermodynamic model and analysis for strand displacement probe/strand displacement blocker system.

The reactions in the MT solution were,

$$\mathrm{MT}+\mathrm{BL} \underset{}{\overset{K_{\mathrm{BM}}}{\rightleftharpoons}} \mathrm{BMT}+\mathrm{L}$$

$$\mathrm{MT}+\mathrm{PS} \underset{}{\overset{K_{\mathrm{PM}}}{\rightleftharpoons}} \mathrm{PMT}+\mathrm{S}$$

The reactions in the WT solution were,

$$\mathrm{WT}+\mathrm{BL} \underset{}{\overset{K_{\mathrm{BW}}}{\rightleftharpoons}} \mathrm{BWT}+\mathrm{L}$$

$$\mathrm{WT}+\mathrm{PS} \underset{}{\overset{K_{\mathrm{PW}}}{\rightleftharpoons}} \mathrm{PWT}+\mathrm{S}$$

According to the mass-action equilibria,

$$\frac{[\mathrm{BMT}][\mathrm{L}]}{[\mathrm{MT}][\mathrm{BL}]}=K_{\mathrm{BM}} \quad (52) \qquad \frac{[\mathrm{BWT}][\mathrm{L}]}{[\mathrm{WT}][\mathrm{BL}]}=K_{\mathrm{BW}} \quad (53)$$

$$\frac{[\mathrm{PMT}][\mathrm{S}]}{[\mathrm{MT}][\mathrm{PS}]}=K_{\mathrm{PM}} \quad (54) \qquad \frac{[\mathrm{PWT}][\mathrm{S}]}{[\mathrm{WT}][\mathrm{PS}]}=K_{\mathrm{PW}} \quad (55)$$

$$[\mathrm{MT}]+[\mathrm{PMT}]+[\mathrm{BMT}]=c_0 \quad (56) \qquad [\mathrm{WT}]+[\mathrm{PWT}]+[\mathrm{BWT}]=c_0 \quad (57)$$

$$[\mathrm{PMT}]+[\mathrm{PS}]=[\mathrm{PS}]_0 \quad (58) \qquad [\mathrm{PWT}]+[\mathrm{PS}]=[\mathrm{PS}]_0 \quad (59)$$

$$[\mathrm{S}]+[\mathrm{PMT}]=[\mathrm{S}]_0 \quad (60) \qquad [\mathrm{S}]+[\mathrm{PWT}]=[\mathrm{S}]_0 \quad (61)$$

$$[\mathrm{BMT}]+[\mathrm{BL}]=[\mathrm{BL}]_0 \quad (62) \qquad [\mathrm{BWT}]+[\mathrm{BL}]=[\mathrm{BL}]_0 \quad (63)$$

$$[\mathrm{L}]+[\mathrm{BMT}]=[\mathrm{L}]_0 \quad (64) \qquad [\mathrm{L}]+[\mathrm{BWT}]=[\mathrm{L}]_0 \quad (65)$$

Combining equations above, we could obtain the expression of $[\mathrm{PMT}]$ and $[\mathrm{PWT}]$.

$$[\mathrm{PMT}]=\frac{[\mathrm{PS}][\mathrm{L}]K_{\mathrm{PM}}c_0}{[\mathrm{BL}][\mathrm{S}]K_{\mathrm{BM}}+[\mathrm{PS}][\mathrm{S}]K_{\mathrm{PM}}+[\mathrm{L}][\mathrm{S}]} \quad (66)$$

$$[\mathrm{PWT}]=\frac{[\mathrm{PS}][\mathrm{L}]K_{\mathrm{PW}}c_0}{[\mathrm{BL}][\mathrm{S}]K_{\mathrm{BW}}+[\mathrm{PS}][\mathrm{L}]K_{\mathrm{PW}}+[\mathrm{L}][\mathrm{S}]} \quad (67)$$

Then we adopted the following approximations:

$$[\mathrm{S}]=[\mathrm{S}]_0,\ [\mathrm{L}]=[\mathrm{L}]_0+c_0,\ [\mathrm{BL}]=[\mathrm{BL}]_0-c_0,\ [\mathrm{PS}]=[\mathrm{PS}]_0-c_0$$

We could obtain the analytic expressions of sensitivity and specificity,

$$\mathrm{Sensitivity}=\frac{\left([\mathrm{PS}]_0-c_0\right)\left([\mathrm{L}]_0+c_0\right)K_{\mathrm{PM}}}{\left([\mathrm{BL}]_0-c_0\right)[\mathrm{S}]_0K_{\mathrm{BM}}+\left([\mathrm{PS}]_0-c_0\right)\left([\mathrm{L}]_0+c_0\right)K_{\mathrm{PM}}+\left([\mathrm{L}]_0+c_0\right)[\mathrm{L}]_0} \quad (68)$$

$$\mathrm{Specificity}=\frac{K_{\mathrm{PM}}\left(\left([\mathrm{BL}]_0-c_0\right)[\mathrm{S}]_0K_{\mathrm{BW}}+\left([\mathrm{PS}]_0-c_0\right)\left([\mathrm{L}]_0+c_0\right)K_{\mathrm{PW}}+\left([\mathrm{L}]_0+c_0\right)[\mathrm{S}]_0\right)}{K_{\mathrm{PW}}\left(\left([\mathrm{BL}]_0-c_0\right)[\mathrm{S}]_0K_{\mathrm{BM}}+\left([\mathrm{PS}]_0-c_0\right)\left([\mathrm{L}]_0+c_0\right)K_{\mathrm{PM}}+\left([\mathrm{L}]_0+c_0\right)[\mathrm{S}]_0\right)} \quad (69)$$

The relations between the involved free energy changes were as follows,

$$\Delta G_{\mathrm{PM}}=\Delta G_{\mathrm{PW}}-\Delta\Delta G_{\mathrm{P}} \quad (70)$$

$$\Delta G_{\mathrm{BM}}=\Delta G_{\mathrm{BW}}+\Delta\Delta G_{\mathrm{B}} \quad (71)$$

The analytic expressions of sensitivity and specificity could be further transformed into,

$$\text{Sensitivity} = \frac{\left([\text{PS}]_0 - c_0\right)\left([\text{L}]_0 + c_0\right)\text{e}^{-\frac{\Delta G_{\text{PM}}}{RT}}}{\left([\text{BL}]_0 - c_0\right)[\text{S}]_0\,\text{e}^{-\frac{\Delta G_{\text{BW}} + \Delta\Delta G_{\text{B}}}{RT}} + \left([\text{PS}]_0 - c_0\right)\left([\text{L}]_0 + c_0\right)\text{e}^{-\frac{\Delta G_{\text{PM}}}{RT}} + \left([\text{L}]_0 + c_0\right)[\text{S}]_0} \tag{72}$$

$$\text{Specificity} = \text{e}^{\frac{\Delta\Delta G_{\text{P}}}{RT}} \times \frac{\left([\text{BL}]_0 - c_0\right)[\text{S}]_0\,\text{e}^{-\frac{\Delta G_{\text{BW}}}{RT}} + \left([\text{PS}]_0 - c_0\right)\left([\text{L}]_0 + c_0\right)\text{e}^{-\frac{\Delta G_{\text{PM}} + \Delta\Delta G_{\text{P}}}{RT}} + \left([\text{L}]_0 + c_0\right)[\text{S}]_0}{\left([\text{BL}]_0 - c_0\right)[\text{S}]_0\,\text{e}^{-\frac{\Delta G_{\text{BW}} + \Delta\Delta G_{\text{B}}}{RT}} + \left([\text{PS}]_0 - c_0\right)\left([\text{L}]_0 + c_0\right)\text{e}^{-\frac{\Delta G_{\text{PM}}}{RT}} + \left([\text{L}]_0 + c_0\right)[\text{S}]_0} \tag{73}$$

Since,

$$\mu = [\text{BL}]K_{\text{BW}} = \left([\text{BL}]_0 - c_0\right)\text{e}^{-\frac{\Delta G_{\text{BW}}}{RT}} \tag{74}$$

Thus,

$$\text{Sensitivity} = \frac{\left([\text{PS}]_0 - c_0\right)\left([\text{L}]_0 + c_0\right)\text{e}^{-\frac{\Delta G_{\text{PM}}}{RT}}}{\mu[\text{S}]_0\,\text{e}^{-\frac{\Delta\Delta G_{B}}{RT}} + \left([\text{PS}]_0 - c_0\right)\left([\text{L}]_0 + c_0\right)\text{e}^{-\frac{\Delta G_{\text{PM}}}{RT}} + \left([\text{L}]_0 + c_0\right)[\text{S}]_0} \tag{75}$$

$$\text{Specificity} = \text{e}^{\frac{\Delta\Delta G_{\text{P}}}{RT}} \times \frac{\mu[\text{S}]_0 + \left([\text{PS}]_0 - c_0\right)\left([\text{L}]_0 + c_0\right)\text{e}^{-\frac{\Delta G_{\text{PM}} + \Delta\Delta G_{\text{P}}}{RT}} + \left([\text{L}]_0 + c_0\right)[\text{S}]_0}{\mu[\text{S}]_0\,\text{e}^{-\frac{\Delta\Delta G_{\text{B}}}{RT}} + \left([\text{PS}]_0 - c_0\right)\left([\text{L}]_0 + c_0\right)\text{e}^{-\frac{\Delta G_{\text{PM}}}{RT}} + \left([\text{L}]_0 + c_0\right)[\text{S}]_0} \tag{76}$$

Derivation of sensitivity,

$$\frac{\partial\text{Sensitivity}}{\partial[\text{BL}]_0} = \frac{d\text{Sensitivity}}{d\mu} \times \frac{\partial\mu}{\partial[\text{BL}]_0} = \frac{\left(c_0 - [\text{PS}]_0\right)\left([\text{L}]_0 + c_0\right)[\text{S}]_0\,\text{e}^{-\frac{\Delta G_{\text{PM}} + \Delta\Delta G_{\text{B}}}{RT}}}{\left(\left([\text{PS}]_0 - c_0\right)\left([\text{L}]_0 + c_0\right)\text{e}^{-\frac{\Delta G_{\text{PM}}}{RT}} + [\text{S}]_0\left(\mu\text{e}^{-\frac{\Delta\Delta G_{\text{B}}}{RT}} + [\text{L}]_0 + c_0\right)\right)^2} \times \text{e}^{-\frac{\Delta G_{\text{BW}}}{RT}} \tag{77}$$

$$\frac{\partial\text{Sensitivity}}{\partial\left(-\Delta G_{\text{BW}}\right)} = \frac{d\text{Sensitivity}}{d\mu} \times \frac{\partial\mu}{\partial\left(-\Delta G_{\text{BW}}\right)} = \frac{\left(c_0 - [\text{PS}]_0\right)\left([\text{L}]_0 + c_0\right)[\text{S}]_0\,\text{e}^{-\frac{\Delta G_{\text{PM}} + \Delta\Delta G_{\text{B}}}{RT}}}{\left(\left([\text{PS}]_0 - c_0\right)\left([\text{L}]_0 + c_0\right)\text{e}^{-\frac{\Delta G_{\text{PM}}}{RT}} + [\text{S}]_0\left(\mu\text{e}^{-\frac{\Delta\Delta G_{\text{B}}}{RT}} + [\text{L}]_0 + c_0\right)\right)^2} \times \frac{([\text{BL}]_0 - c_0)\text{e}^{-\frac{\Delta G_{\text{BW}}}{RT}}}{RT} \tag{78}$$

Since,

$$\Delta\Delta G_{\text{P}} > 0,\quad \Delta\Delta G_{\text{B}} > 0,\quad c_0 - [\text{PS}]_0 < 0 \tag{79}$$

Therefore,

$$\frac{\partial\text{Sensitivity}}{\partial[\text{BL}]_0} < 0 \;,\; \frac{\partial\text{Sensitivity}}{\partial\left(-\Delta G_{\text{BW}}\right)} < 0 \tag{80}$$

Thus, the sensitivity is monotonically decreasing over $[\text{B}]_0$ and $-\Delta G_{\text{BW}}$.

For the derivation of specificity,

$$\frac{\partial\text{Specificity}}{\partial[\text{BL}]_0} = \frac{d\text{Specificity}}{d\mu} \times \frac{\partial\mu}{\partial[\text{BL}]_0} = \frac{\left([\text{L}]_0 + c_0\right)[\text{S}]_0\left(\left([\text{PS}]_0 - c_0\right)\left(\text{e}^{-\frac{\Delta G_{\text{PM}} - \Delta\Delta G_{\text{P}}}{RT}} - \text{e}^{-\frac{\Delta G_{\text{PM}} + \Delta\Delta G_{\text{B}}}{RT}}\right) + [\text{S}]_0\left(\text{e}^{\frac{\Delta\Delta G_{\text{P}}}{RT}} - \text{e}^{\frac{\Delta\Delta G_{\text{P}} - \Delta\Delta G_{\text{B}}}{RT}}\right)\right)}{\left(\left([\text{PS}]_0 - c_0\right)\left([\text{L}]_0 + c_0\right)\text{e}^{-\frac{\Delta G_{\text{PM}}}{RT}} + [\text{S}]_0\left(\mu\text{e}^{-\frac{\Delta\Delta G_{\text{B}}}{RT}} + [\text{L}]_0 + c_0\right)\right)^2} \times \text{e}^{-\frac{\Delta G_{\text{BW}}}{RT}} \tag{81}$$

$$\frac{\partial \text{Specificity}}{\partial(-\Delta G_{\text{BW}})} = \frac{d\text{Specificity}}{d\mu} \times \frac{\partial \mu}{\partial(-\Delta G_{\text{BW}})} = \frac{\left([\text{L}]_0 + c_0\right)[\text{S}]_0 \left(\left([\text{PS}]_0 - c_0\right)\left(\text{e}^{-\frac{\Delta G_{\text{PM}} - \Delta\Delta G_{\text{P}}}{RT}} - \text{e}^{-\frac{\Delta G_{\text{PM}} + \Delta\Delta G_{\text{B}}}{RT}}\right) + [\text{S}]_0 \left(\text{e}^{\frac{\Delta\Delta G_{\text{P}}}{RT}} - \text{e}^{\frac{\Delta\Delta G_{\text{P}} - \Delta\Delta G_{\text{B}}}{RT}}\right)\right)}{\left(\left([\text{PS}]_0 - c_0\right)\left([\text{L}]_0 + c_0\right)\text{e}^{-\frac{\Delta G_{\text{PM}}}{RT}} + [\text{S}]_0 \left(\mu \text{e}^{-\frac{\Delta\Delta G_{\text{B}}}{RT}} + [\text{L}]_0 + c_0\right)\right)^2} \times \frac{\left([\text{BL}]_0 - c_0\right)\text{e}^{-\frac{\Delta G_{\text{BW}}}{RT}}}{RT} \tag{82}$$

Then,

$$\frac{\partial \text{Specificity}}{\partial [\text{BL}]_0} > 0 \ , \ \frac{\partial \text{Specificity}}{\partial(-\Delta G_{\text{BW}})} > 0 \tag{83}$$

Thus, the specificity is monotonically increasing over $[\text{B}]_0$ and $-\Delta G_{\text{BW}}$.

$$\lim_{\mu \to 0} \text{Sensitivity} = \frac{\left([\text{PS}]_0 - c_0\right)\left([\text{L}]_0 + c_0\right)\text{e}^{-\frac{\Delta G_{\text{PM}}}{RT}}}{\left([\text{PS}]_0 - c_0\right)\left([\text{L}]_0 + c_0\right)\text{e}^{-\frac{\Delta G_{\text{PM}}}{RT}} + \left([\text{L}]_0 + c_0\right)[\text{S}]_0} \quad (\text{Maximum}) \tag{84}$$

$$\lim_{\mu \to 0} \text{Specificity} = \text{e}^{\frac{\Delta\Delta G_{\text{P}}}{RT}} \times \frac{\left([\text{PS}]_0 - c_0\right)\left([\text{L}]_0 + c_0\right)\text{e}^{-\frac{\Delta G_{PM} + \Delta\Delta G_{\text{P}}}{RT}} + \left([\text{L}]_0 + c_0\right)[\text{S}]_0}{\left([\text{PS}]_0 - c_0\right)\left([\text{L}]_0 + c_0\right)\text{e}^{-\frac{\Delta G_{PM}}{RT}} + \left([\text{L}]_0 + c_0\right)[\text{S}]_0} \quad (\text{Minimum}) \tag{85}$$

$$\lim_{\mu \to +\infty} \text{Sensitivity} = 0 \quad (\text{Minimum}) \tag{86}$$

$$\lim_{\mu \to +\infty} \text{Specificity} = \text{e}^{\frac{\Delta\Delta G_{\text{P}}}{RT}} \times \text{e}^{\frac{\Delta\Delta G_{\text{B}}}{RT}} \quad (\text{Maximum}) \tag{87}$$

To sum up,

$$\text{Sensitivity}([\text{BL}]_0, -\Delta G_{\text{BW}}) \in [\frac{([\text{PS}]_0 - c_0)([\text{L}]_0 + c_0)\text{e}^{-\frac{\Delta G_{\text{PM}}}{RT}}}{([\text{PS}]_0 - c_0)([\text{L}]_0 + c_0)\text{e}^{-\frac{\Delta G_{\text{PM}}}{RT}} + ([\text{L}]_0 + c_0)[\text{S}]_0}, 0) \searrow \tag{88}$$

$$\text{Specificity}([\text{BL}]_0, -\Delta G_{\text{BW}}) \in [\text{e}^{\frac{\Delta\Delta G_{\text{P}}}{RT}} \times \frac{([\text{PS}]_0 - c_0)([\text{L}]_0 + c_0)\text{e}^{-\frac{\Delta G_{\text{PM}} + \Delta\Delta G_{\text{P}}}{RT}} + ([\text{L}]_0 + c_0)[\text{S}]_0}{([\text{PS}]_0 - c_0)([\text{L}]_0 + c_0)\text{e}^{-\frac{\Delta G_{\text{PM}}}{RT}} + ([\text{L}]_0 + c_0)[\text{S}]_0}, \text{e}^{\frac{\Delta\Delta G_{\text{P}}}{RT}} \times \text{e}^{\frac{\Delta\Delta G_{\text{B}}}{RT}}) \nearrow \tag{89}$$

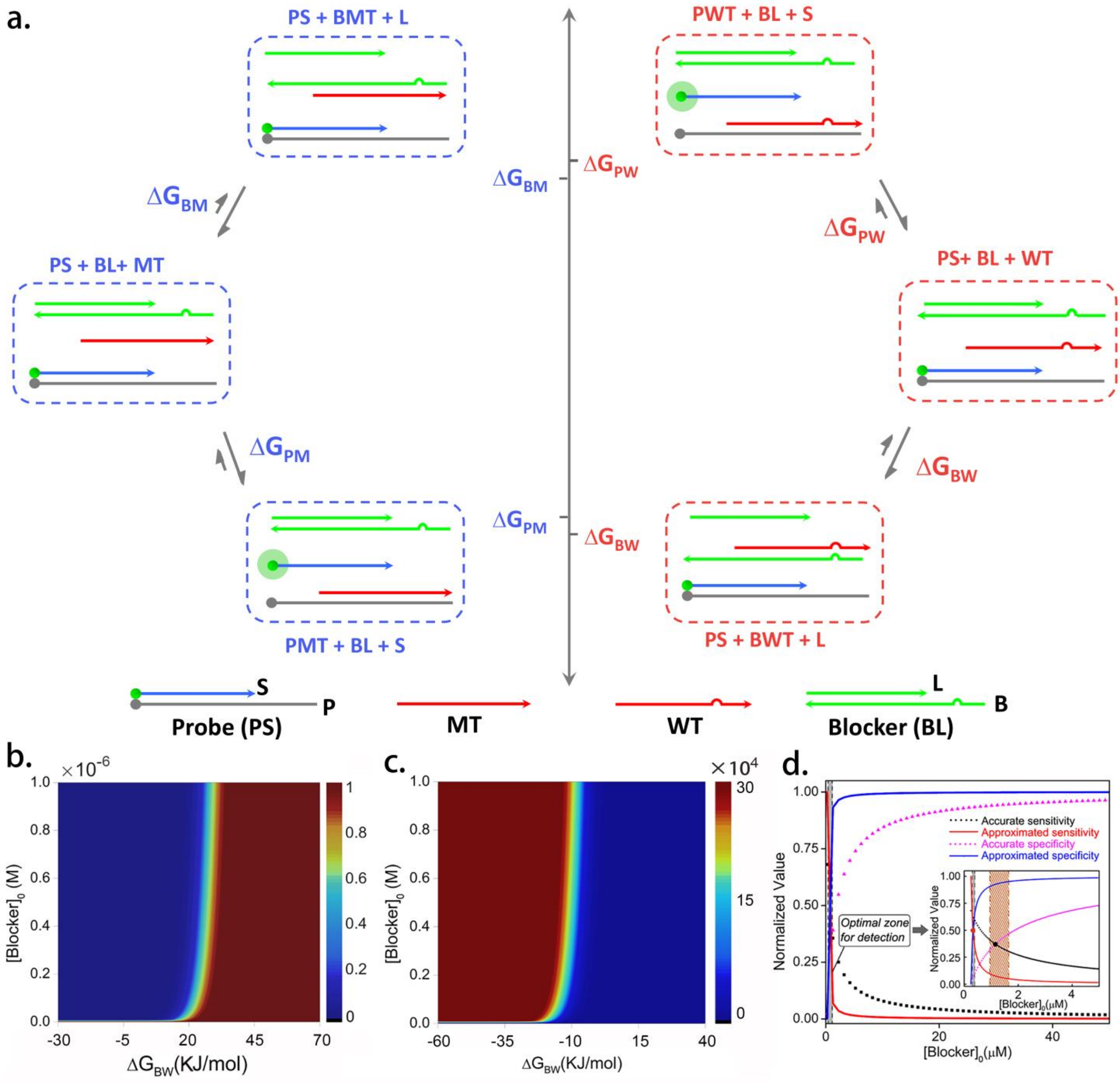

Supplementary Figure 1. Thermodynamic model of the Strand displacement probe/strand displacement blocker system. (a) The reaction pathways and levels of associated free energy changes. (b) The calculated sensitivity of the Strand displacement probe/blocker system. (c) The calculated specificity of the Strand displacement probe/blocker system over $[\text{Blocker}]_0$ (also denoted as $[\text{BLBS}]_0$) and -ΔG_{BW}. (d) Two-dimensional interception curves of sensitivity and specificity by setting -ΔG_{BW} at a fixed value of -11522 J/mol.

Supplementary Note 3.

Thermodynamic model and analysis for strand displacement probe/standard blocker system.

The reactions in the MT solution were,

$$\mathrm{MT}+\mathrm{B} \underset{}{\overset{K_{\mathrm{BM}}}{\rightleftharpoons}} \mathrm{BMT}$$

$$\mathrm{MT}+\mathrm{PS} \underset{}{\overset{K_{\mathrm{PM}}}{\rightleftharpoons}} \mathrm{PMT}+\mathrm{S}$$

$$\mathrm{S}+\mathrm{B} \underset{}{\overset{K_{\mathrm{BS}}}{\rightleftharpoons}} \mathrm{BS}$$

The reactions in the WT solution were,

$$\mathrm{WT}+\mathrm{B} \underset{}{\overset{K_{\mathrm{BW}}}{\rightleftharpoons}} \mathrm{BWT}$$

$$\mathrm{WT}+\mathrm{PS} \underset{}{\overset{K_{\mathrm{PW}}}{\rightleftharpoons}} \mathrm{PWT}+\mathrm{S}$$

$$\mathrm{S}+\mathrm{B} \underset{}{\overset{K_{\mathrm{BS}}}{\rightleftharpoons}} \mathrm{BS}$$

According to the mass-action equilibria,

$$\frac{[\mathrm{BMT}]}{[\mathrm{MT}][\mathrm{B}]}=K_{\mathrm{BM}} \quad (90) \qquad \frac{[\mathrm{BWT}]}{[\mathrm{WT}][\mathrm{B}]}=K_{\mathrm{BW}} \quad (91)$$

$$\frac{[\mathrm{PMT}][\mathrm{S}]}{[\mathrm{MT}][\mathrm{PS}]}=K_{\mathrm{PM}} \quad (92) \qquad \frac{[\mathrm{PWT}][\mathrm{S}]}{[\mathrm{WT}][\mathrm{PS}]}=K_{\mathrm{PW}} \quad (93)$$

$$\frac{[\mathrm{BS}]}{[\mathrm{S}][\mathrm{B}]}=K_{\mathrm{BS}} \quad (94) \qquad \frac{[\mathrm{BS}]}{[\mathrm{S}][\mathrm{B}]}=K_{\mathrm{BS}} \quad (95)$$

$$[\mathrm{MT}]+[\mathrm{PMT}]+[\mathrm{BMT}]=\mathrm{c}_0 \quad (96) \qquad [\mathrm{WT}]+[\mathrm{PWT}]+[\mathrm{BWT}]=c_0 \quad (97)$$

$$[\mathrm{PMT}]+[\mathrm{PS}]=[\mathrm{PS}]_0 \quad (98) \qquad [\mathrm{PWT}]+[\mathrm{PS}]=[\mathrm{PS}]_0 \quad (99)$$

$$[\mathrm{BMT}]+[\mathrm{B}]+[\mathrm{BS}]=[\mathrm{B}]_0 \quad (100) \qquad [\mathrm{BWT}]+[\mathrm{B}]+[\mathrm{BS}]=[\mathrm{B}]_0 \quad (101)$$

$$[\mathrm{S}]+[\mathrm{BS}]-[\mathrm{PMT}]=[\mathrm{S}]_0 \quad (102) \qquad [\mathrm{S}]+[\mathrm{BS}]-[\mathrm{PWT}]=[\mathrm{S}]_0 \quad (103)$$

Combine equations above, we could have,

$$[\mathrm{BS}]=\frac{K_{\mathrm{PM}}K_{\mathrm{BC}}}{K_{\mathrm{BM}}}\times\frac{K_{\mathrm{BM}}[\mathrm{B}]\left([\mathrm{PMT}]-[\mathrm{PS}]_0\right)\left([\mathrm{PMT}]-c_0\right)}{[\mathrm{PMT}]\left([\mathrm{B}]K_{\mathrm{BM}}+1\right)} \quad (104)$$

$$[\mathrm{S}]=\frac{K_{\mathrm{PM}}\left([\mathrm{PMT}]-[\mathrm{PS}]_0\right)\left([\mathrm{PMT}]-c_0\right)}{[\mathrm{PMT}]\left([\mathrm{B}]K_{\mathrm{BM}}+1\right)} \quad (105)$$

Then, we could obtain the quadric equation in $[\mathrm{PMT}]$,

$$[\mathrm{PMT}]+[\mathrm{S}]_0=\frac{K_{\mathrm{PM}}K_{\mathrm{BC}}}{K_{\mathrm{BM}}}\times\frac{K_{\mathrm{BM}}[\mathrm{B}]\left([\mathrm{PS}]_0-[\mathrm{PMT}]\right)\left(c_0-[\mathrm{PMT}]\right)}{[\mathrm{PMT}]\left([\mathrm{B}]K_{\mathrm{BM}}+1\right)}+\frac{K_{\mathrm{PM}}\left([\mathrm{PS}]_0-[\mathrm{PMT}]\right)\left(c_0-[\mathrm{PMT}]\right)}{[\mathrm{PMT}]\left([\mathrm{B}]K_{\mathrm{BM}}+1\right)} \quad (106)$$

Similarly, we could obtain the quadric equation in $[\mathrm{PWT}]$.

$$[\mathrm{PWT}]+[\mathrm{S}]_0=\frac{K_{\mathrm{PW}}K_{\mathrm{BC}}}{K_{\mathrm{BW}}}\times\frac{K_{\mathrm{BW}}[\mathrm{B}]\left([\mathrm{PS}]_0-[\mathrm{PWT}]\right)\left(c_0-[\mathrm{PWT}]\right)}{[\mathrm{PWT}]\left([\mathrm{B}]K_{\mathrm{BW}}+1\right)}+\frac{K_{\mathrm{PW}}\left([\mathrm{PS}]_0-[\mathrm{PWT}]\right)\left(c_0-[\mathrm{PWT}]\right)}{[\mathrm{PWT}]\left([\mathrm{B}]K_{\mathrm{BW}}+1\right)} \quad (107)$$

The relations between the involved free energy changes were as follows,

$$\Delta G_{\mathrm{PM}}=\Delta G_{\mathrm{PW}}-\Delta\Delta G_{\mathrm{P}} \quad (108)$$

$$\Delta G_{\mathrm{BM}}=\Delta G_{\mathrm{BW}}+\Delta\Delta G_{\mathrm{B}} \quad (109)$$

Combine the above two quadric equations, we could obtain analytic expressions of sensitivity over μ_{BM},

$$\text{Sensitivity}=\frac{\left([\mathrm{PS}]_0+c_0\right)\frac{\frac{K_{\mathrm{PM}}K_{\mathrm{BM}}}{K_{\mathrm{BS}}}\mu_{\mathrm{BM}}+K_{\mathrm{PW}}}{\mu_{\mathrm{BM}}+1}+[\mathrm{S}]_0-\sqrt{\left([\mathrm{PS}]_0-c_0\right)^2\left(\frac{\frac{K_{\mathrm{PM}}K_{\mathrm{BM}}}{K_{\mathrm{BS}}}\mu_{\mathrm{BM}}+K_{\mathrm{PW}}}{\mu_{\mathrm{BM}}+1}\right)^2+\left(4[\mathrm{PS}]_0c_0+2[\mathrm{PS}]_0[\mathrm{S}]_0+2[\mathrm{S}]_0c_0\right)\frac{\frac{K_{\mathrm{PM}}K_{\mathrm{BM}}}{K_{\mathrm{BS}}}\mu_{\mathrm{BM}}+K_{\mathrm{PW}}}{\mu_{\mathrm{BM}}+1}+[\mathrm{S}]_0^2}}{2c_0\left(\frac{\frac{K_{\mathrm{PM}}K_{\mathrm{BM}}}{K_{\mathrm{BS}}}\mu_{\mathrm{BM}}+K_{\mathrm{PW}}}{\mu_{\mathrm{BM}}+1}-1\right)} \tag{110}$$

Where,

$$\mu_{\mathrm{BM}}=[\mathrm{B}]K_{\mathrm{BM}}=\left([\mathrm{B}]_0-c_0\right)\mathrm{e}^{-\frac{\Delta G_{\mathrm{BM}}}{RT}}=\mathrm{e}^{-\frac{\Delta\Delta G_{\mathrm{B}}}{RT}}\mu \tag{111}$$

Define the sensitivity of a toehold probe alone toward targeting strand as $F(x)$, in which the variable x was the equilibrium constant,

Then, for PS + T→ PT + S,

$$\frac{[\mathrm{PT}][\mathrm{S}]}{[\mathrm{PS}][\mathrm{T}]}=x \tag{112}$$

Also,

$$[\mathrm{S}]=[\mathrm{S}]_0+[\mathrm{PT}] \tag{113}$$

$$[\mathrm{PS}]=[\mathrm{PS}]_0-[\mathrm{PT}] \tag{114}$$

$$[\mathrm{T}]=c_0-[\mathrm{PT}] \tag{115}$$

Combine the above four equations, we have

$$F\left(x,[\mathrm{PS}]_0\right)=\frac{[\mathrm{PT}]}{c_0}=\frac{\left([\mathrm{PS}]_0+c_0\right)x+[\mathrm{S}]_0-\sqrt{\left([\mathrm{PS}]_0-c_0\right)^2x^2+\left(4[\mathrm{PS}]_0c_0+2[\mathrm{PS}]_0[\mathrm{S}]_0+2[\mathrm{S}]_0c_0\right)x+[\mathrm{S}]_0^2}}{2c_0\left(x-1\right)} \tag{116}$$

In our experiments, the $[\mathrm{PS}]_0$ was constant when tuning the blocker sequence and concentration, so the binary function of $F(x, [\mathrm{PS}]_0)$ could be simplified as a unary function of $F(x)$.

Then, the expression of sensitivity could be simplified as,

$$\text{Sensitivity}=F\left(\frac{\frac{K_{\mathrm{PM}}K_{\mathrm{BS}}}{K_{\mathrm{BM}}}\mu_{\mathrm{BM}}+K_{\mathrm{PM}}}{\mu_{\mathrm{BM}}+1}\right)=F\left(\frac{\mathrm{e}^{-\frac{\Delta G_{\mathrm{PM}}-\Delta G_{\mathrm{BW}}+\Delta G_{\mathrm{BS}}}{RT}}\mu+\mathrm{e}^{-\frac{\Delta G_{\mathrm{PM}}}{RT}}}{\mathrm{e}^{-\frac{\Delta\Delta G_B}{RT}}\mu+1}\right) \tag{117}$$

Similarly, we could the analytic expression of specificity,

$$\text{Specificity}=\frac{F\left(\frac{\frac{K_{\mathrm{PM}}K_{\mathrm{BS}}}{K_{\mathrm{BM}}}\mu_{\mathrm{BM}}+K_{\mathrm{PM}}}{\mu_{\mathrm{BM}}+1}\right)}{F\left(\frac{\frac{K_{\mathrm{PW}}K_{\mathrm{BS}}}{K_{\mathrm{BW}}}\mu+K_{\mathrm{PW}}}{\mu+1}\right)}=\frac{F\left(\frac{\mathrm{e}^{-\frac{\Delta G_{\mathrm{PM}}-\Delta G_{\mathrm{BW}}+\Delta G_{\mathrm{BS}}}{RT}}\mu+\mathrm{e}^{-\frac{\Delta G_{\mathrm{PM}}}{RT}}}{e^{-\frac{\Delta\Delta G_B}{RT}}\mu+1}\right)}{F\left(\frac{\mathrm{e}^{-\frac{\Delta G_{\mathrm{PM}}+\Delta\Delta G_{\mathrm{P}}-\Delta G_{\mathrm{BW}}+\Delta G_{\mathrm{BS}}}{RT}}\mu+\mathrm{e}^{-\frac{\Delta G_{\mathrm{PW}}}{RT}}}{\mu+1}\right)} \tag{118}$$

To figure out the monotonicity of sensitivity and specificity, we firstly needed to demonstrate the monotonicity of $F(x)$,

$$\frac{dF(x)}{dx}=\frac{P-Q}{2c_0\left(x-1\right)^2\sqrt{\left([\mathrm{PS}]_0-c_0\right)^2x^2+\left(4[\mathrm{PS}]_0c_0+2[\mathrm{S}]_0c_0+2[\mathrm{PS}]_0[\mathrm{S}]_0\right)x+[\mathrm{S}]_0^2}} \tag{119}$$

Where,

$$P=\left([\mathrm{S}]_0c_0+[\mathrm{PS}]_0[\mathrm{S}]_0+c_0^2+[\mathrm{PS}]_0^2\right)x+2[\mathrm{PS}]_0c_0+[\mathrm{S}]_0c_0+[\mathrm{PS}]_0[\mathrm{S}]_0+[\mathrm{S}]_0^2 \tag{120}$$

$$Q=\left([\mathrm{PS}]_0+c_0+[\mathrm{S}]_0\right)\sqrt{\left([\mathrm{PS}]_0-c_0\right)^2x^2+\left(4[\mathrm{PS}]_0c_0+2[\mathrm{S}]_0c_0+2[\mathrm{PS}]_0[\mathrm{S}]_0\right)x+[\mathrm{S}]_0^2} \tag{121}$$

Since,

$$P^2-Q^2=c_0\left(x-1\right)^2\left([\mathrm{PS}]_0+[\mathrm{S}]_0\right)\left([\mathrm{S}]_0+c_0\right)[\mathrm{PS}]_0>0 \tag{122}$$

Thus,

$$P > Q \tag{123}$$

$$\frac{dF\left(x\right)}{dx} > 0 \tag{124}$$

Then, we could calculate the monotonicity of sensitivity and specificity,

$$\frac{\partial \text{Sensitivity}}{\partial [\text{B}]_0} = \frac{dF(x)}{dx} \times \frac{d(\frac{\frac{K_{\text{PM}}K_{\text{BS}}}{K_{BM}}\mu_{\text{BM}} + K_{\text{PM}}}{\mu_{BM} + 1})}{d\mu_{\text{BM}}} \times \frac{\partial \mu_{\text{BM}}}{\partial([\text{B}]_0)} \tag{125}$$

$$\frac{d(\frac{\frac{K_{\text{PM}}K_{\text{BS}}}{K_{\text{BM}}}\mu_{\text{BM}} + K_{\text{PM}}}{\mu_{\text{BM}} + 1})}{d\mu_{\text{BM}}} = \frac{K_{\text{PM}}(K_{\text{BS}} - K_{\text{BM}})}{K_{\text{BS}}\left(\mu_{\text{BM}} + 1\right)^2} < 0 \tag{126}$$

$$\frac{\partial \mu_{\text{BM}}}{\partial([\text{B}]_0)} = K_{\text{BM}} > 0 \tag{127}$$

Thus,

$$\frac{\partial \text{Sensitivity}}{\partial [\text{B}]_0} < 0 \tag{128}$$

Also,

$$\frac{\partial \text{Sensitivity}}{\partial(-\Delta G_{\text{BW}})} = \frac{dF(x)}{dx} \times \frac{d(\frac{\frac{K_{\text{PM}}K_{\text{BS}}}{K_{\text{BM}}}\mu_{\text{BM}} + K_{\text{PM}}}{\mu_{\text{BM}} + 1})}{d\mu_{\text{BM}}} \times \frac{\partial \mu_{\text{BM}}}{\partial(-\Delta G_{\text{BW}})} < 0 \tag{129}$$

Thus, the sensitivity is monotonically decreasing over $[\text{B}]_0$ and $-\Delta G_{\text{BW}}$.

$$\text{Sensitivity}([\text{B}]_0, -\Delta G_{\text{BW}}) \in [F(\text{e}^{-\frac{\Delta G_{\text{PM}}}{RT}}), F(\text{e}^{-\frac{\Delta G_{\text{PM}} - \Delta G_{\text{BM}} - \Delta\Delta G_{\text{B}} + \Delta G_{\text{BS}}}{RT}})) \searrow \tag{130}$$

For specificity, we found that it was not always monotonically increasing over $[\text{B}]_0$ and $-\Delta G_{\text{BW}}$ under all occasions. For certain thermodynamic parameters, the specificity curve could present small fluctuations when varying $[\text{B}]_0$. But the amplitude of the fluctuations was so tiny that as a whole, the specificity seemed to be increasing over $[\text{B}]_0$ and $-\Delta G_{\text{BW}}$. It was worth noting that for the modelling probe (probe-3) of Figure 2d, the specificity was rigorously increasing over $[\text{B}]_0$ and $-\Delta G_{\text{BW}}$.

We then could calculate the limit of specificity,

$$\lim_{\mu_{BW} \to 0} \text{Specificity} = \frac{F(\text{e}^{-\frac{\Delta G_{\text{PM}}}{RT}})}{F(\text{e}^{-\frac{\Delta G_{\text{PM}} + \Delta\Delta G_{\text{P}}}{RT}})} \quad \text{(quasi minimum)} \tag{131}$$

$$\lim_{\mu_{BW} \to +\infty} \text{Specificity} = \frac{F(\text{e}^{-\frac{\Delta G_{\text{PM}} - \Delta G_{\text{BW}} - \Delta\Delta G_{\text{B}} + \Delta G_{\text{BS}}}{RT}})}{F(\text{e}^{-\frac{\Delta G_{\text{PM}} + \Delta\Delta G_{\text{P}} - \Delta G_{\text{BW}} + \Delta G_{\text{BS}}}{RT}})} \quad \text{(quasi maximum)} \tag{132}$$

Therefore, the quasi value range of specificity was,

$$\text{Specificity}([\text{B}]_0, -\Delta G_{\text{BW}}) \in [\frac{F(\text{e}^{-\frac{\Delta G_{\text{PM}}}{RT}})}{F(\text{e}^{-\frac{\Delta G_{\text{PM}} + \Delta\Delta G_{\text{P}}}{RT}})}, \frac{F(\text{e}^{-\frac{\Delta G_{\text{PM}} - \Delta G_{\text{BW}} - \Delta\Delta G_{\text{B}} + \Delta G_{\text{BS}}}{RT}})}{F(\text{e}^{-\frac{\Delta G_{\text{PM}} + \Delta\Delta G_{P} - \Delta G_{\text{BW}} + \Delta G_{\text{BS}}}{RT}})}) \tag{133}$$

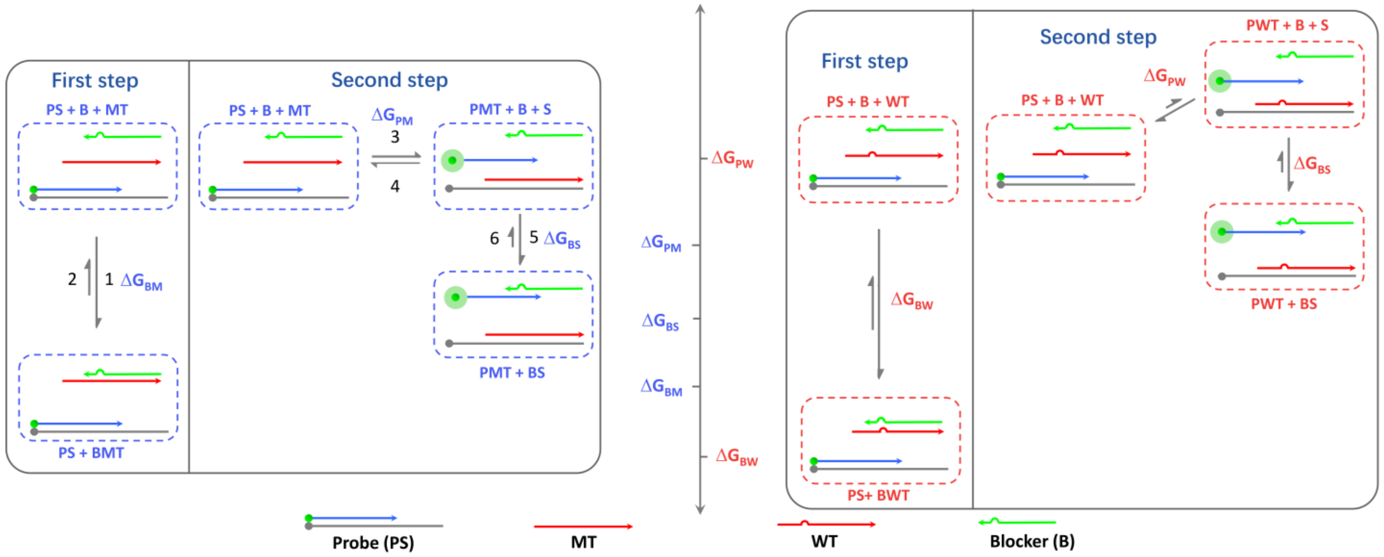

Supplementary Figure 2. The reaction pathways and levels of associated free energy changes in the Strand displacement probe/standard blocker system.

As was discussed in the main article, the above thermodynamic model required the Strand displacement probe/standard blocker system to reach thermodynamic equilibrium, which was impossible within several hours. Therefore, the actual reaction pathways were as follows,

The thermodynamic equilibriums of first step and second step were independent and would not interfere with each other. Therefore, according to the mass-action equilibria,

$$[\mathrm{MT}]_{\mathrm{step1}} = \frac{c_0}{1+K_{\mathrm{BM}}[\mathrm{B}]} \tag{134}$$

For the second step, we have,

$$\frac{[\mathrm{PMT}][\mathrm{S}]}{[\mathrm{MT}][\mathrm{PS}]} = K_{\mathrm{PM}} \tag{135}$$

$$\frac{[\mathrm{PWT}][\mathrm{S}]}{[\mathrm{WT}][\mathrm{PS}]} = K_{\mathrm{PW}} \tag{136}$$

$$\frac{[\mathrm{BS}]}{[\mathrm{S}][\mathrm{B}]} = K_{\mathrm{BS}} \tag{137}$$

$$\frac{[\mathrm{BS}]}{[\mathrm{S}][\mathrm{B}]} = K_{\mathrm{BS}} \tag{138}$$

$$[\mathrm{MT}]+[\mathrm{PMT}] = \frac{c_0}{1+K_{\mathrm{BM}}[\mathrm{B}]} \tag{139}$$

$$[\mathrm{WT}]+[\mathrm{PWT}] = \frac{c_0}{1+K_{\mathrm{BW}}[\mathrm{B}]} \tag{140}$$

$$[\mathrm{PS}] = [\mathrm{PS}]_0 \tag{141}$$

$$[\mathrm{PS}] = [\mathrm{PS}]_0 \tag{142}$$

$$[\mathrm{B}] = [\mathrm{B}]_0 - c_0 \tag{143}$$

$$[\mathrm{B}] = [\mathrm{B}]_0 - c_0 \tag{144}$$

$$[\mathrm{S}]+[\mathrm{BS}]-[\mathrm{PMT}] = [\mathrm{S}]_0 \tag{145}$$

$$[\mathrm{S}]+[\mathrm{BS}]-[\mathrm{PWT}] = [\mathrm{S}]_0 \tag{146}$$

Combining the above equations, we could obtain,

$$[\mathrm{PMT}] = \frac{\sqrt{\left(K_{\mathrm{PM}}[\mathrm{PS}]_0(([\mathrm{B}]_0-c_0)K_{\mathrm{BS}}+1)+[\mathrm{S}]_0\right)^2+4c_0\times\frac{1+K_{\mathrm{BS}}([\mathrm{B}]_0-c_0)}{1+K_{\mathrm{BM}}([\mathrm{B}]_0-c_0)}}-\left(K_{PM}[\mathrm{PS}]_0(([\mathrm{B}]_0-c_0)K_{\mathrm{BS}}+1)+[\mathrm{S}]_0\right)}{2} \tag{148}$$

Similarly,

$$[\mathrm{PWT}] = \frac{\sqrt{\left(K_{\mathrm{PW}}[\mathrm{PS}]_0(([\mathrm{B}]_0-c_0)K_{\mathrm{BC}}+1)+[\mathrm{S}]_0\right)^2+4c_0\times\frac{1+K_{\mathrm{BS}}([\mathrm{B}]_0-c_0)}{1+K_{\mathrm{BW}}([\mathrm{B}]_0-c_0)}}-\left(K_{\mathrm{PW}}[\mathrm{PS}]_0(([\mathrm{B}]_0-c_0)K_{\mathrm{BS}}+1)+[\mathrm{S}]_0\right)}{2} \tag{149}$$

Introducing the relevant parameters of the modelling probe (Probe-3) into the above equations, we could draw the interception curves of the sensitivity and specificity over $[\mathrm{B}]_0$.

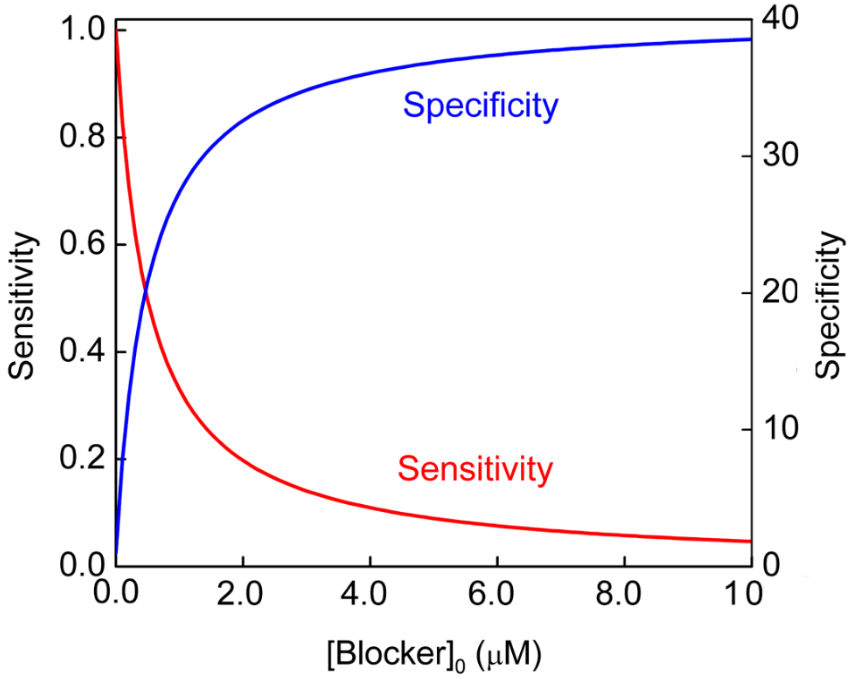

Supplementary Figure 3. The predicted sensitivity and specificity of Strand displacement probe/standard blocker system. The equilibriums of reactions 1-2 and reactions 3-6 were assumed to be independent.

Modelling results showed that as $[B]_0$ increased, the sensitivity decreased to around 0 whereas the specificity increased to the upper limit, in accordance with the experimental results.

Supplementary Note 4.

Thermodynamic model and analysis for dissociative 4-way strand-exchange led competitive DNA testing system.

The reactions in the MT solution were,

$$\mathrm{MT}+\mathrm{B} \underset{}{\overset{K_{\mathrm{BM}}}{\rightleftharpoons}} \mathrm{BMT}$$

$$\mathrm{MT}+\mathrm{PS} \underset{}{\overset{K_{\mathrm{PM}}}{\rightleftharpoons}} \mathrm{PMT}+\mathrm{S}$$

$$\mathrm{PS}+\mathrm{BMT} \underset{}{\overset{K_{\mathrm{PBM}}}{\rightleftharpoons}} \mathrm{PMT}+\mathrm{BS}$$

The reactions in the WT solution were,

$$\mathrm{WT}+\mathrm{B} \underset{}{\overset{K_{\mathrm{BW}}}{\rightleftharpoons}} \mathrm{BWT}$$

$$\mathrm{WT}+\mathrm{PS} \underset{}{\overset{K_{\mathrm{PW}}}{\rightleftharpoons}} \mathrm{PWT}+\mathrm{S}$$

$$\mathrm{PS}+\mathrm{BWT} \underset{}{\overset{K_{\mathrm{PBW}}}{\rightleftharpoons}} \mathrm{PWT}+\mathrm{BS}$$

According to the mass-action equilibria,

$$\frac{[\mathrm{BMT}]}{[\mathrm{MT}][\mathrm{B}]}=K_{\mathrm{BM}} \quad (150)$$

$$\frac{[\mathrm{BWT}]}{[\mathrm{WT}][\mathrm{B}]}=K_{\mathrm{BW}} \quad (151)$$

$$\frac{[\mathrm{PMT}][\mathrm{S}]}{[\mathrm{MT}][\mathrm{PS}]}=K_{\mathrm{PM}} \quad (152)$$

$$\frac{[\mathrm{PWT}][\mathrm{S}]}{[\mathrm{WT}][\mathrm{PS}]}=K_{\mathrm{PW}} \quad (153)$$

$$\frac{[\mathrm{BS}][\mathrm{PMT}]}{[\mathrm{PS}][\mathrm{BMT}]}=K_{\mathrm{PBM}} \quad (154)$$

$$\frac{[\mathrm{BS}][\mathrm{PWT}]}{[\mathrm{PS}][\mathrm{BWT}]}=K_{\mathrm{PBW}} \quad (155)$$

$$[\mathrm{MT}]+[\mathrm{PMT}]+[\mathrm{BMT}]=c_0 \quad (156)$$

$$[\mathrm{WT}]+[\mathrm{PWT}]+[\mathrm{BWT}]=c_0 \quad (157)$$

$$[\mathrm{PMT}]+[\mathrm{PS}]=[\mathrm{PS}]_0 \quad (158)$$

$$[\mathrm{PWT}]+[\mathrm{PS}]=[\mathrm{PS}]_0 \quad (159)$$

$$[\mathrm{BMT}]+[\mathrm{B}]+[\mathrm{BS}]=[\mathrm{B}]_0 \quad (160)$$

$$[\mathrm{BWT}]+[\mathrm{B}]+[\mathrm{BS}]=[\mathrm{B}]_0 \quad (161)$$

$$[\mathrm{S}]+[\mathrm{BS}]-[\mathrm{PMT}]=[\mathrm{S}]_0 \quad (162)$$

$$[\mathrm{S}]+[\mathrm{BS}]-[\mathrm{PWT}]=[\mathrm{S}]_0 \quad (163)$$

Combining equations above, we could have,

$$[\mathrm{BS}]=K_{\mathrm{PBM}}\times\frac{K_{\mathrm{BM}}[\mathrm{B}]\left([\mathrm{PS}]_0-[\mathrm{PMT}]\right)\left(c_0-[\mathrm{PMT}]\right)}{[\mathrm{PMT}]\left([\mathrm{B}]K_{\mathrm{BM}}+1\right)} \quad (164)$$

$$[\mathrm{S}]=\frac{K_{\mathrm{PM}}\left([\mathrm{PS}]_0-[\mathrm{PMT}]\right)\left(c_0-[\mathrm{PMT}]\right)}{[\mathrm{PMT}]\left([\mathrm{B}]K_{\mathrm{BM}}+1\right)} \quad (165)$$

Then, we could obtain the quadric equation in $[\mathrm{PMT}]$,

$$[\mathrm{PMT}]+[\mathrm{S}]_0=K_{\mathrm{PBM}}\times\frac{K_{\mathrm{BM}}[\mathrm{B}]\left([\mathrm{PS}]_0-[\mathrm{PMT}]\right)\left(c_0-[\mathrm{PMT}]\right)}{[\mathrm{PMT}]\left([\mathrm{B}]K_{\mathrm{BM}}+1\right)}+\frac{K_{\mathrm{PM}}\left([\mathrm{PS}]_0-[\mathrm{PMT}]\right)\left(c_0-[\mathrm{PMT}]\right)}{[\mathrm{PMT}]\left([\mathrm{B}]K_{\mathrm{BM}}+1\right)} \quad (166)$$

Similarly, we could obtain the quadric equation in $[\mathrm{PWT}]$.

$$[\mathrm{PWT}]+[\mathrm{S}]_0=K_{\mathrm{PBW}}\times\frac{K_{\mathrm{BW}}[\mathrm{B}]\left([\mathrm{PS}]_0-[\mathrm{PWT}]\right)\left(c_0-[\mathrm{PWT}]\right)}{[\mathrm{PWT}]\left([\mathrm{B}]K_{\mathrm{BW}}+1\right)}+\frac{K_{\mathrm{PW}}\left([\mathrm{PS}]_0-[\mathrm{PWT}]\right)\left(c_0-[\mathrm{PWT}]\right)}{[\mathrm{PWT}]\left([\mathrm{B}]K_{\mathrm{BW}}+1\right)} \quad (167)$$

The relations between the involved free energy changes were as follows,

$$\Delta G_{\mathrm{PBM}}=\Delta G_{\mathrm{PM}} \quad (168)$$

$$\Delta G_{\mathrm{PW}} - \Delta G_{\mathrm{PM}} = \Delta\Delta G_{\mathrm{P}} \tag{169}$$

$$\Delta G_{\mathrm{PBW}} - \Delta G_{\mathrm{PBM}} = \Delta\Delta G_{\mathrm{P}} + \Delta\Delta G_{\mathrm{B}} \tag{170}$$

Combine the above two quadric equations, we could obtain analytic expressions of sensitivity and specificity over μ,

$$\mathrm{Sensitivity} = \frac{\left([\mathrm{PS}]_0 + c_0\right)K_{\mathrm{PM}} + [\mathrm{S}]_0 - \sqrt{\left([\mathrm{PS}]_0 - c_0\right)^2 K_{\mathrm{PM}}{}^2 + \left(4[\mathrm{PS}]_0 c_0 + 2[\mathrm{PS}]_0[\mathrm{S}]_0 + 2[\mathrm{S}]_0 c_0\right)K_{\mathrm{PM}} + [\mathrm{S}]_0{}^2}}{2c_0\left(K_{\mathrm{PM}} - 1\right)} \tag{171}$$

$$\mathrm{Specificity} = \frac{\left(\frac{K_{\mathrm{PBW}}\mu_{\mathrm{BM}} + K_{\mathrm{PW}}}{\mu_{\mathrm{BM}} + 1} - 1\right) \times \left(\left([\mathrm{PS}]_0 + c_0\right)K_{\mathrm{PM}} + [\mathrm{S}]_0 - \sqrt{\left([\mathrm{PS}]_0 - c_0\right)^2 K_{\mathrm{PM}}{}^2 + \left(4[\mathrm{PS}]_0 c_0 + 2[\mathrm{PS}]_0[\mathrm{S}]_0 + 2[\mathrm{S}]_0 c_0\right)K_{\mathrm{PM}} + [\mathrm{S}]_0{}^2}\right)}{\left(K_{\mathrm{PM}} - 1\right) \times \left(\left([\mathrm{PS}]_0 + c_0\right)\frac{K_{\mathrm{PBW}}\mu_{\mathrm{BM}} + K_{\mathrm{PW}}}{\mu_{\mathrm{BM}} + 1} + [\mathrm{S}]_0 - \sqrt{\left([\mathrm{PS}]_0 - c_0\right)^2 \left(\frac{K_{\mathrm{PBW}}\mu_{\mathrm{BM}} + K_{\mathrm{PW}}}{\mu_{\mathrm{BM}} + 1}\right)^2 + (4[\mathrm{PS}]_0 c_0 + 2[\mathrm{PS}]_0[S]_0 + 2[\mathrm{S}]_0 c_0)\frac{K_{\mathrm{PBW}}\mu_{\mathrm{BM}} + K_{\mathrm{PW}}}{\mu_{\mathrm{BM}} + 1} + [\mathrm{S}]_0{}^2}\right)} \tag{172}$$

Then, the expression of sensitivity could be simplified as,

$$\mathrm{Sensitivity} = F\left(K_{\mathrm{PM}}\right) = F(\mathrm{e}^{-\frac{\Delta G_{\mathrm{PM}}}{RT}}) \equiv \mathrm{constant} \tag{173}$$

$$\mathrm{Specificity} = \frac{F(K_{\mathrm{PM}})}{F(\frac{K_{\mathrm{PBW}}\mu + K_{\mathrm{PW}}}{\mu + 1})} = \frac{F(\mathrm{e}^{-\frac{\Delta G_{\mathrm{PM}}}{RT}})}{F(\frac{\mathrm{e}^{-\frac{\Delta G_{\mathrm{BW}} + \Delta G_{\mathrm{PBW}}}{RT}}([\mathrm{B}]_0 - c_0) + \mathrm{e}^{-\frac{\Delta G_{\mathrm{PW}}}{RT}}}{\mathrm{e}^{-\frac{\Delta G_{\mathrm{BW}}}{RT}}([\mathrm{B}]_0 - c_0) + 1})} \tag{174}$$

Note that for the expression of sensitivity, no approximations were adopted. We could prove that the specificity monotonically increasing over [B] and K_{BW},

$$\frac{d\mathrm{Specificity}}{d[\mathrm{B}]} = \frac{d\mathrm{Specificity}}{dF(\frac{K_{\mathrm{PBW}}\mu + K_{\mathrm{PW}}}{\mu + 1})} \times \frac{dF(\frac{K_{\mathrm{PBW}}\mu + K_{\mathrm{PW}}}{\mu + 1})}{d\mu} \times \frac{d\mu}{d[\mathrm{B}]} \tag{175}$$

$$\frac{d\mathrm{Specificity}}{dF(\frac{K_{\mathrm{PBW}}\mu + K_{\mathrm{PW}}}{\mu + 1})} = -\frac{F(K_{\mathrm{PM}})}{F^2(\frac{K_{\mathrm{PBW}}\mu + K_{\mathrm{PW}}}{\mu + 1})} < 0 \tag{176}$$

Since $K_{\mathrm{PBW}} < K_{\mathrm{PW}}$,

$$\frac{dF(\frac{K_{\mathrm{PBW}}\mu + K_{\mathrm{PW}}}{\mu + 1})}{d\mu} = \frac{K_{\mathrm{PBW}} - K_{\mathrm{PW}}}{(\mu + 1)^2} < 0 \tag{177}$$

$$\frac{d\mu}{d[\mathrm{B}]} = K_{\mathrm{BW}} > 0 \tag{178}$$

Therefore,

$$\frac{d\mathrm{Specificity}}{d[\mathrm{B}]} > 0 \tag{179}$$

Furthermore, we could prove the monotonicity of [PWT] and specificity over K_{BW} through the proof by contradiction described above,

Consider an increase in K_{BW}, we want to prove that [PWT] necessarily decreases.

For contradiction, assume not, $[\mathrm{PWT}] \geq [\mathrm{PWT_{old}}]$ (180)

Since, $[\mathrm{PWT}] + [\mathrm{PS}] = [\mathrm{PS}]_0 \equiv \mathrm{constant}$ (181)

Thus, $[\mathrm{PS}] \leq [\mathrm{PS_{old}}]$ (182)

Then,

$$[\mathrm{WT}] = \frac{[\mathrm{PWT}][\mathrm{S}]}{[\mathrm{PS}]K_{\mathrm{PW}}},\ [\mathrm{BWT}] = \frac{[\mathrm{PWT}][\mathrm{BS}]}{[\mathrm{PS}]K_{\mathrm{PBW}}}$$
$$[\mathrm{WT}]K_{\mathrm{PW}} + [\mathrm{BWT}]K_{\mathrm{PBW}} = \frac{[\mathrm{PWT}]([\mathrm{S}]+[\mathrm{BS}])}{[\mathrm{PS}]} = \frac{[\mathrm{PWT}]([\mathrm{PWT}]+[\mathrm{S}]_0)}{[\mathrm{PS}]} \geq [\mathrm{WT_{old}}]K_{\mathrm{PW}} + [\mathrm{BWT_{old}}]K_{\mathrm{PBW}} \tag{183}$$

Also,

$$[\mathrm{WT}]+[\mathrm{BWT}] = c_0 - [\mathrm{PWT}] \leq [\mathrm{WT_{old}}]+[\mathrm{BWT_{old}}] \tag{184}$$

Combine the above two equations,

$$\frac{[\mathrm{WT_{old}}]K_{\mathrm{PW}} + [\mathrm{BWT_{old}}]K_{\mathrm{PBW}} - [\mathrm{WT}]K_{\mathrm{PW}}}{K_{\mathrm{PBW}}} \leq [\mathrm{BWT}] \leq [\mathrm{WT_{old}}]+[\mathrm{BWT_{old}}]-[\mathrm{WT}]$$
$$\frac{K_{\mathrm{PW}}}{K_{\mathrm{PBW}}}([\mathrm{WT_{old}}]-[\mathrm{WT}]) \leq [\mathrm{WT_{old}}]-[\mathrm{WT}] \tag{185}$$

Since $K_{\mathrm{PW}} > K_{\mathrm{PBW}}$ Then, $[\mathrm{WT_{old}}]-[\mathrm{WT}] \leq 0$ (186)

Similarly,

$$\frac{[\mathrm{WT_{old}}]K_{\mathrm{PW}} + [\mathrm{BWT_{old}}]K_{\mathrm{PBW}} - [\mathrm{BWT}]K_{\mathrm{PBW}}}{K_{\mathrm{PW}}} \leq [\mathrm{WT}] \leq [\mathrm{WT_{old}}]+[\mathrm{BWT_{old}}]-[\mathrm{BWT}]$$
$$\frac{K_{\mathrm{PBW}}}{K_{\mathrm{PW}}}([\mathrm{BWT_{old}}]-[\mathrm{BWT}]) \leq [\mathrm{BWT_{old}}]-[\mathrm{BWT}] \tag{187}$$
$$[\mathrm{BWT_{old}}]-[\mathrm{BWT}] \geq 0$$

On the other side,

$$\frac{[\mathrm{S}]}{K_{\mathrm{PW}}} + \frac{[\mathrm{BS}]}{K_{\mathrm{PBW}}} = \frac{([\mathrm{WT}]+[\mathrm{BWT}])[\mathrm{PS}]}{[\mathrm{PWT}]} = \frac{([\mathrm{S}]_0 - [\mathrm{PWT}])[\mathrm{PS}]}{[PWT]} \leq \frac{[\mathrm{S_{old}}]}{K_{\mathrm{PW}}} + \frac{[\mathrm{BS_{old}}]}{K_{\mathrm{PBW}}}$$
$$[\mathrm{S}]+[\mathrm{BS}] = c_0 + [\mathrm{PWT}] \geq [\mathrm{S_{old}}]+[\mathrm{BS_{old}}] \tag{188}$$

Then,

$$\frac{[\mathrm{S_{old}}]}{K_{\mathrm{PW}}} + \frac{[\mathrm{BS_{old}}]}{K_{\mathrm{PBW}}} - \frac{[\mathrm{BS}]}{K_{\mathrm{PBW}}} \geq \frac{[S]}{K_{\mathrm{PW}}} \geq \frac{[\mathrm{S_{old}}]+[\mathrm{BS_{old}}]-[\mathrm{BS}]}{K_{\mathrm{PBW}}}$$
$$\frac{[\mathrm{BS_{old}}]-[\mathrm{BS}]}{K_{\mathrm{PBW}}} \geq \frac{[\mathrm{BS_{old}}]-[\mathrm{BS}]}{K_{\mathrm{PW}}} \tag{189}$$
$$[\mathrm{BS_{old}}]-[\mathrm{BS}] \geq 0$$

Since, $[\mathrm{BWT}] \leq [\mathrm{BWT_{old}}],\ [\mathrm{BS}] \leq [\mathrm{BS_{old}}]$ (190)

$$[\mathrm{B}] = [\mathrm{B}]_0 - [\mathrm{BS}] - [\mathrm{BWT}] \geq [\mathrm{B_{old}}] \tag{191}$$

Overall, we have $[\mathrm{BWT}] \leq [\mathrm{BWT_{old}}],\ [\mathrm{B}] \geq [\mathrm{B_{old}}],\ [\mathrm{WT}] \geq [\mathrm{WT_{old}}]$, which lead to

$$K_{\mathrm{BW}} = \frac{[\mathrm{BWT}]}{[\mathrm{WT}][\mathrm{B}]} \leq \frac{[\mathrm{BWT_{old}}]}{[\mathrm{WT_{old}}][\mathrm{B_{old}}]} = K_{\mathrm{BW_{old}}} \tag{192}$$

The above equation is contradictory to the initial condition of an increase in K_{BW}. Conclusively, we have proved that the sensitivity is monotonically increasing over [B] and K_{BW}.

Then, the value ranges of sensitivity and specificity could be determined,

$$\mathrm{Sensitivity}([\mathrm{B}],\ K_{\mathrm{BW}}) = F(\mathrm{e}^{-\frac{\Delta G_{\mathrm{PM}}}{RT}}) \equiv \mathrm{constant} \tag{193}$$

$$\text{Specificity}([\text{B}], K_{\text{BW}}) \in [\frac{F(\mathrm{e}^{-\frac{\Delta G_{\text{PM}}}{RT}})}{F(\mathrm{e}^{-\frac{\Delta G_{\text{PW}}}{RT}})}, \frac{F(\mathrm{e}^{-\frac{\Delta G_{\text{PM}}}{RT}})}{F(\mathrm{e}^{-\frac{\Delta G_{\text{PBW}}}{RT}})}) \nearrow \tag{194}$$

However, we could not judge the monotonicity of [PWT] over $[\text{Blocker}]_0$ due to the extremely complexity of the expressions. Therefore, we adopted the approximations of $[\text{B}]=[\text{B}]_0\text{-}c_0$. Then,

$$\frac{\partial \text{Specificity}}{\partial [\text{B}]_0} = \frac{d\text{Specificity}}{dF(\frac{K_{\text{PBW}}\mu + K_{\text{PW}}}{\mu+1})} \times \frac{dF(\frac{K_{\text{PBW}}\mu + K_{\text{PW}}}{\mu+1})}{d\mu} \times \frac{\partial \mu}{\partial [\text{B}]_0} \tag{195}$$

Since,

$$\frac{\partial \text{Specificity}}{\partial [\text{B}]_0} > 0 \tag{196}$$

Thus,

$$\frac{\partial \text{Specificity}}{\partial (-\Delta G_{\text{BW}})} = \frac{d\text{Specificity}}{dF(\frac{K_{\text{PBW}}\mu + K_{\text{PW}}}{\mu+1})} \times \frac{dF(\frac{K_{\text{PBW}}\mu + K_{\text{PW}}}{\mu+1})}{d\mu} \times \frac{\partial \mu}{\partial (-\Delta G_{\text{BW}})} > 0 \tag{197}$$

The specificity is monotonically decreasing over $[\text{B}]_0$ and $-\Delta G_{\text{BW}}$.

$$\lim_{\mu \to 0} \text{Specificity} = \frac{F(K_{\text{PM}})}{F(K_{\text{PW}})} = \frac{F(\mathrm{e}^{-\frac{\Delta G_{\text{PM}}}{RT}})}{F(\mathrm{e}^{-\frac{\Delta G_{\text{PM}}+\Delta\Delta G_{\text{P}}}{RT}})} \quad (\text{Minimum}) \tag{198}$$

$$\lim_{\mu \to \infty} \text{Specificity} = \frac{F(K_{\text{PM}})}{F(K_{\text{PBW}})} = \frac{F(\mathrm{e}^{-\frac{\Delta G_{\text{PM}}}{RT}})}{F(\mathrm{e}^{-\frac{\Delta G_{\text{PM}}+\Delta\Delta G_{\text{P}}+\Delta\Delta G_{\text{B}}}{RT}})} \quad (\text{Maximum}) \tag{199}$$

To sum up,

$$\text{Sensitivity}([\text{B}]_0, -\Delta G_{\text{BW}}) = F(\frac{\mu_{\text{BM}}K_{\text{PBM}} + K_{\text{PM}}}{\mu_{\text{BM}}+1}) = F(\mathrm{e}^{-\frac{\Delta G_{\text{PM}}}{RT}}) \equiv \text{constant} \tag{200}$$

$$\text{Specificity}([\text{B}]_0, -\Delta G_{\text{BW}}) = \frac{F(K_{\text{PM}})}{F(\frac{\mu K_{\text{PBW}} + K_{\text{PW}}}{\mu+1})} \in [\frac{F(\mathrm{e}^{-\frac{\Delta G_{\text{PM}}}{RT}})}{F(\mathrm{e}^{-\frac{\Delta G_{\text{PM}}+\Delta\Delta G_{\text{P}}}{RT}})}, \frac{F(\mathrm{e}^{-\frac{\Delta G_{\text{PM}}}{RT}})}{F(\mathrm{e}^{-\frac{\Delta G_{\text{PM}}+\Delta\Delta G_{\text{P}}+\Delta\Delta G_{\text{B}}}{RT}})}) \nearrow \tag{201}$$

Supplementary Note 5.

Discussion on the essential contribution of the 4-Way SELECT system.

As was discussed in the results section of the main article, equation (10) and (11) has revealed that the 4-Way SELECT system could be equivalent to a single toehold probe with its equilibrium constants toward MT and WT regulated by blocker. Based on this, we could explain the essential contribution of the 4-Way SELECT system had made to the field. For de novo design of a probe/blocker competitive composition system, although the 4-Way SELECT system still suffered from the inverse correlation over probe, it had distinct advantages against the conventional systems:

i) If we compared equations (10)-(11) with equations (3)-(4), we could see that the conventional competitive system suffered from the inverse correlation over both blocker and probe, so the optimization burden was in the order of n^2. Whereas, for the dissociative 4-Way SELECT system, it only suffered from the inverse correlation over probe, and we only needed to optimize the probe design. The optimization burden was in the order of n, much smaller than that of conventional systems.

ii) More importantly, the design of probe sequence and concentration for the 4-Way SELECT system could be experimentally free. In real applications, we certainly would use long and excessive blocker strands to make the system function around its best specificity. Under such set-ups, the whole 4-Way SELECT system was equivalent to a single toehold probe alone with equilibrium constants of K_{PBM} (identical to K_{PM1}) and K_{PBW}, and the corresponding sensitivity and specificity of the single toehold probe and the 4-Way SELECT system were $F(K_{\mathrm{PM1}})$ and $F(K_{\mathrm{PM1}})/F(K_{\mathrm{PBW}})$. Since the function of $F(x)$ was derived without taking any approximations at all, $F(K_{\mathrm{PM1}})$ and $F(K_{\mathrm{PM1}})/F(K_{\mathrm{PBW}})$ were highly accurate and could be used for computerized de novo design of the 4-Way SELECT system without any experimental optimization at all. However, for conventional competitive systems, thermodynamic modelling and computer work were far from enough: First, as was discussed in the results section, the large deviations within the optimal zone made the theoretical prediction highly inaccurate. Second, the conventional competitive system was not able to reach thermodynamic equilibrium due to the ultra-slow kinetics of dsDNA dissociation. For example, the blocker and probe with same lengths of 18-nt and same concentrations would produce a sensitivity around 0.5 according to the thermodynamic model. However, the dissociation rate of blocker off the targeting strand was extremely small, so we could only observe a very low sensitivity (nearly 0), which was much deviated from the predicted value of ~0.5. It was worth noting that the kinetics was not a problem for the 4-Way SELECT system, as was thoroughly discussed in Figure 4. To sum up, the large deviations within the optimal detection zone in combination with the ultra-slow kinetics made the quantitative prediction for conventional competitive composition systems highly inaccurate, and thereby intensive experimental optimization was inevitable. Whereas, owing to the unique thermodynamics and fast kinetics, de novo design of the dissociative 4-Way SELECT system could be totally computerized and experimentally free.

iii) Since $F(K_{\mathrm{PM1}})$ and $F(K_{\mathrm{PM1}})/F(K_{\mathrm{PBW}})$ were completely accurate with no approximations at all, empirical rules could be applied to further eliminate the computer work. Many published papers have reported that for systems consisting of only probes and MT/WT, the near-optimal performance could be achieved simply by setting $\Delta G_{\mathrm{PM}} \approx 0$. In real applications, the 4-Way SELECT system was equivalent to a system consisting of only toehold probe and MT/WT. Therefore, such empirical rule of $\Delta G_{\mathrm{PM}} \approx 0$ could be applied to our system to make it completely optimization free, as was experimentally demonstrated in Figure 5. We also would like to point out that for dissociative 4-Way SELECT system, $\Delta G_{\mathrm{PM}} \equiv 0$, so it was set to be optimization free by default.

For enhancing the performance of detection systems that already comprised probes: In many cases, researchers may have designed and synthesized strand displacement probes in their assays. But the experimental results, especially the specificity, were not satisfying. To improve the specificity, addition of a blocker strand into the system would be the first choice. In such situations, blockers, designed in the principle of the 4-Way SELECT system, showed huge advantages over other systems: when increasing the specificity of the system, conventional blocker would simultaneously lower down the system's sensitivity, whereas our proposed blocker would not affect the sensitivity at all. Especially for enhancing the specificity of multiplexed assays targeting multiple mutations of interests, our system was extremely convenient to employ and would be the best choice.

Supplementary Note 6.

Thermodynamic model and analysis for non-dissociative mode of 4-way strand-exchange led competitive DNA testing system.

The reactions in the MT solution were,

$$\mathrm{PSMT} \underset{}{\overset{K_{\mathrm{PM}}}{\rightleftharpoons}} \mathrm{PMTS}$$

$$\mathrm{PSBMT} \underset{}{\overset{K_{\mathrm{PBM}}}{\rightleftharpoons}} \mathrm{PMTBS}$$

$$\mathrm{B} + \mathrm{PSMT} \underset{}{\overset{K_{\mathrm{BM}}}{\rightleftharpoons}} \mathrm{PSBMT}$$

The reactions in the WT solution were,

$$\mathrm{PSWT} \underset{}{\overset{K_{\mathrm{PW}}}{\rightleftharpoons}} \mathrm{PWTS}$$

$$\mathrm{PSBWT} \underset{}{\overset{K_{\mathrm{PBW}}}{\rightleftharpoons}} \mathrm{PWTBS}$$

$$\mathrm{B} + \mathrm{PSWT} \underset{}{\overset{K_{\mathrm{BW}}}{\rightleftharpoons}} \mathrm{PSBWT}$$

According to the mass-action equilibria,

$$\frac{[\mathrm{PSBMT}]}{[\mathrm{PSMT}][\mathrm{B}]} = K_{\mathrm{BM}} \quad (202) \qquad \frac{[\mathrm{PSBWT}]}{[\mathrm{PSWT}][\mathrm{B}]} = K_{\mathrm{BW}} \quad (203)$$

$$\frac{[\mathrm{PMTS}]}{[\mathrm{PSMT}]} = K_{\mathrm{PM}} \quad (204) \qquad \frac{[\mathrm{PWTS}]}{[\mathrm{PSWT}]} = K_{\mathrm{PW}} \quad (205)$$

$$\frac{[\mathrm{PMTBS}]}{[\mathrm{PSBMT}]} = K_{\mathrm{PBM}} \quad (206) \qquad \frac{[\mathrm{PWTBS}]}{[\mathrm{PSBWT}]} = K_{\mathrm{PBW}} \quad (207)$$

$$[\mathrm{PSMT}] + [\mathrm{PMTS}] + [\mathrm{PSBMT}] + [\mathrm{PMTBS}] = c_0 \quad (208) \qquad [\mathrm{PSWT}] + [\mathrm{PWTS}] + [\mathrm{PSBWT}] + [\mathrm{PWTBS}] = c_0 \quad (209)$$

$$[\mathrm{B}] + [\mathrm{PSBMT}] + [\mathrm{PMTBS}] = [\mathrm{B}]_0 \quad (210) \qquad [\mathrm{B}] + [\mathrm{PSBWT}] + [\mathrm{PWTBS}] = [\mathrm{B}]_0 \quad (211)$$

Combining equations above, we could have,

$$[\mathrm{PMTS}] = \frac{K_{\mathrm{PM}} c_0}{[\mathrm{B}] K_{\mathrm{BM}} K_{\mathrm{PBM}} + [\mathrm{B}] K_{\mathrm{BM}} + K_{\mathrm{PM}} + 1} \quad (212) \qquad [\mathrm{PWTS}] = \frac{K_{\mathrm{PW}} c_0}{[\mathrm{B}] K_{\mathrm{BW}} K_{\mathrm{PBW}} + [\mathrm{B}] K_{\mathrm{BW}} + K_{\mathrm{PW}} + 1} \quad (213)$$

$$[\mathrm{PMTBS}] = \frac{K_{\mathrm{PBM}} K_{\mathrm{BM}} [\mathrm{B}] c_0}{[\mathrm{B}] K_{\mathrm{BM}} K_{\mathrm{PBM}} + [\mathrm{B}] K_{\mathrm{BM}} + K_{\mathrm{PM}} + 1} \quad (214) \qquad [\mathrm{PWTBS}] = \frac{K_{\mathrm{PBW}} K_{\mathrm{BW}} [\mathrm{B}] c_0}{[\mathrm{B}] K_{\mathrm{BW}} K_{\mathrm{PBW}} + [\mathrm{B}] K_{\mathrm{BW}} + K_{\mathrm{PW}} + 1} \quad (215)$$

Then

$$[\mathrm{PMT}] = [\mathrm{PMTS}] + [\mathrm{PMTBS}] = \frac{K_{\mathrm{PM}} c_0 + K_{\mathrm{PBM}} K_{\mathrm{BM}} [\mathrm{B}] c_0}{[\mathrm{B}] K_{\mathrm{BM}} K_{\mathrm{PBM}} + [\mathrm{B}] K_{\mathrm{BM}} + K_{PM} + 1} \quad (216)$$

$$[\mathrm{PWT}] = [\mathrm{PWTS}] + [\mathrm{PWTBS}] = \frac{K_{\mathrm{PW}} c_0 + K_{\mathrm{PBW}} K_{\mathrm{BW}} [\mathrm{B}] c_0}{[\mathrm{B}] K_{\mathrm{BW}} K_{\mathrm{PBW}} + [\mathrm{B}] K_{\mathrm{BW}} + K_{\mathrm{PW}} + 1} \quad (217)$$

Since,

$$K_{\mathrm{PM}} = K_{\mathrm{PBM}} = 1 \quad (218)$$

Thus,

$$\mathrm{Sensitivity} = \frac{K_{\mathrm{PM}} + K_{\mathrm{PM}} K_{\mathrm{BM}} [\mathrm{B}]}{[\mathrm{B}] K_{\mathrm{BM}} K_{\mathrm{PM}} + [\mathrm{B}] K_{\mathrm{BM}} + K_{\mathrm{PM}} + 1} = \frac{1 + K_{\mathrm{BM}} [\mathrm{B}]}{2 + 2K_{\mathrm{BM}} [\mathrm{B}]} \equiv \frac{1}{2} \quad (219)$$

$$\mathrm{Specificity} = \frac{0.5}{\dfrac{K_{\mathrm{PW}} + \mu K_{\mathrm{PBW}}}{\mu K_{\mathrm{PBW}} + \mu + K_{\mathrm{PW}} + 1}} = \frac{0.5}{\dfrac{\frac{\mu K_{\mathrm{PBW}} + K_{\mathrm{PW}}}{\mu + 1}}{\frac{\mu K_{\mathrm{PBW}} + K_{\mathrm{PW}}}{\mu + 1} + 1}} = \frac{0.5}{G\left(\dfrac{\mu K_{\mathrm{PBW}} + K_{\mathrm{PW}}}{\mu + 1}\right)} \quad (220)$$

Then, we could calculate the monotonicity of specificity,

$$\frac{\partial \text{Specificity}}{\partial [\text{B}]_0} = \frac{d\text{Specificity}}{dG(\frac{K_{\text{PBW}}\mu + K_{\text{PW}}}{\mu+1})} \times \frac{dG(\frac{K_{\text{PBW}}\mu + K_{\text{PW}}}{\mu+1})}{d\mu} \times \frac{\partial \mu}{\partial [\text{B}]_0} \tag{221}$$

$$\frac{d\text{Specificity}}{dG(\frac{K_{\text{PBW}}\mu + K_{\text{PW}}}{\mu+1})} = -\frac{0.5}{G^2(\frac{K_{\text{PBW}}\mu + K_{\text{PW}}}{\mu+1})} < 0 \tag{222}$$

Since $K_{\text{PBW}} < K_{\text{PW}}$,

$$\frac{dG(\frac{K_{\text{PBW}}\mu + K_{\text{PW}}}{\mu+1})}{d\mu} = \frac{K_{\text{PBW}} - K_{\text{PW}}}{(\mu+1)^2} < 0 \tag{223}$$

$$\frac{\partial \mu}{\partial [\text{B}]_0} = K_{\text{BW}} > 0 \tag{224}$$

Thus,

$$\frac{\partial \text{Specificity}}{\partial [\text{B}]_0} > 0 \tag{225}$$

Also,

$$\frac{\partial \text{Specificity}}{\partial (-\Delta G_{\text{BW}})} = \frac{d\text{Specificity}}{dG(\frac{K_{\text{PBW}}\mu + K_{\text{PW}}}{\mu+1})} \times \frac{dG(\frac{K_{\text{PBW}}\mu + K_{\text{PW}}}{\mu+1})}{d\mu} \times \frac{\partial \mu}{\partial (-\Delta G_{\text{BW}})} > 0 \tag{226}$$

Thus, the specificity is monotonically decreasing over $[\text{B}]_0$ and -ΔG_{BW}.

$$\lim_{\mu \to 0} \text{Specificity} = \frac{G(K_{\text{PM}})}{G(K_{\text{PW}})} = \frac{1}{2G(e^{-\frac{\Delta G_{\text{PM}} + \Delta\Delta G_{\text{P}}}{RT}})} \quad \text{(Minimum)} \tag{227}$$

$$\lim_{\mu \to \infty} \text{Specificity} = \frac{G(1)}{G(K_{\text{PBW}})} = \frac{1}{2G(\text{e}^{-\frac{\Delta G_{\text{PM}} + \Delta\Delta G_{\text{P}} + \Delta\Delta G_{\text{B}}}{RT}})} \quad \text{(Maximum)} \tag{228}$$

To sum up,

$$\text{Sensitivity}([\text{B}]_0, -\Delta G_{\text{BW}}) = G(\frac{\mu_{\text{BM}} K_{\text{PBM}} + K_{\text{PM}}}{\mu_{\text{BM}} + 1}) = G(1) \equiv 0.5 \tag{229}$$

$$\text{Specificity}([\text{B}]_0, -\Delta G_{\text{BW}}) = \frac{0.5}{G(\frac{\mu K_{\text{PBW}} + K_{\text{PW}}}{\mu+1})} \in [\frac{1}{2G(\text{e}^{-\frac{\Delta G_{\text{PM}} + \Delta\Delta G_{\text{P}}}{RT}})}, \frac{1}{2G(\text{e}^{-\frac{\Delta G_{\text{PM}} + \Delta\Delta G_{\text{P}} + \Delta\Delta G_{\text{B}}}{RT}})}) \nearrow \tag{230}$$

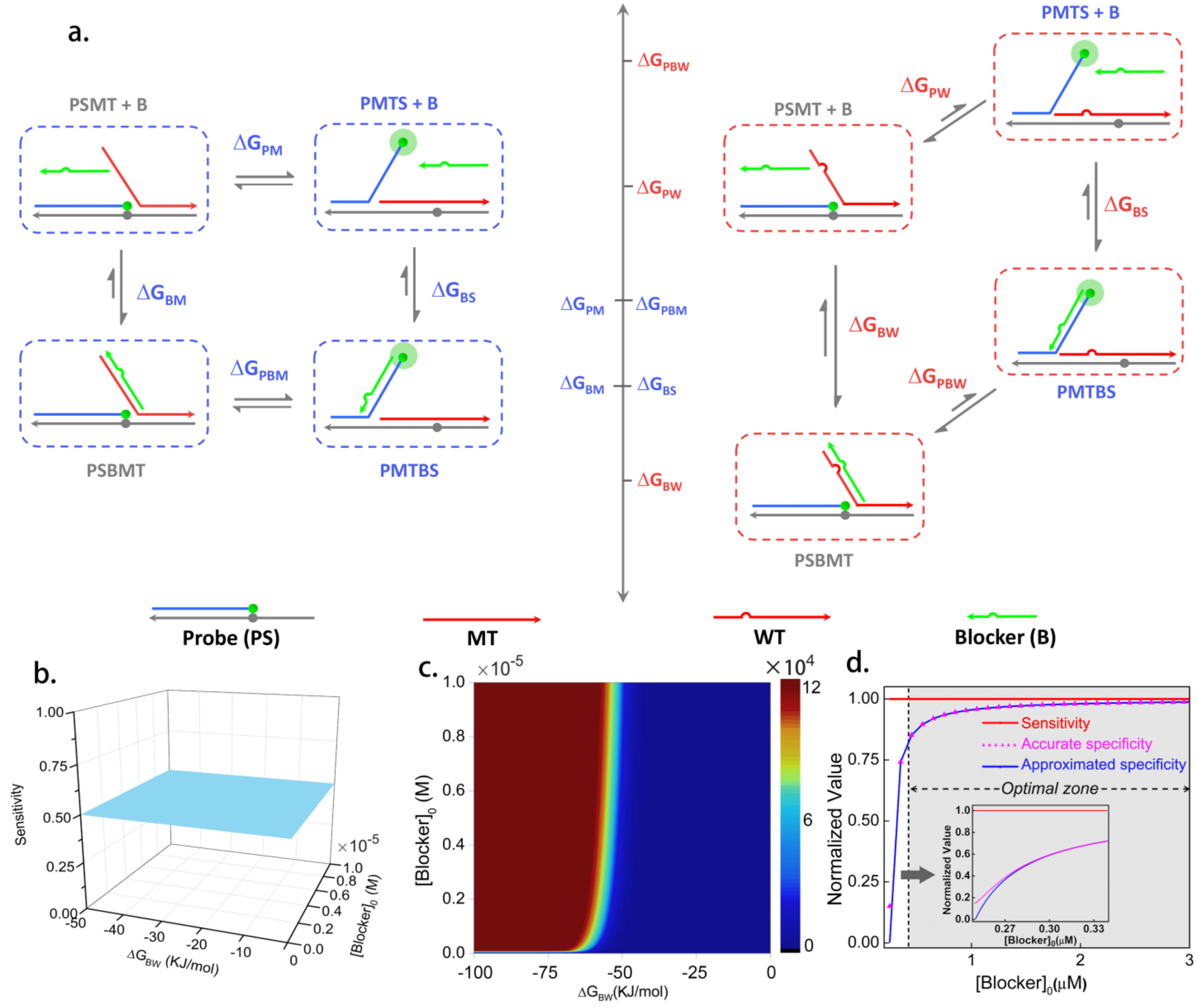

Supplementary Figure 4. Thermodynamic model of the non-dissociative 4-Way SELECT system. (a) The reaction pathways and levels of associated free energy changes in the non-dissociative 4-Way SELECT system. (b) The calculated sensitivity of the non-dissociative 4-Way SELECT system over $[\text{Blocker}]_0$ (also denoted as $[\text{B}]_0$) and $-\Delta G_{\text{BW}}$. (c) The calculated specificity of the non-dissociative 4-Way SELECT system over $[\text{Blocker}]_0$ and $-\Delta G_{\text{BW}}$. (d) Two-dimensional interception curves of sensitivity and specificity by setting $-\Delta G_{\text{BW}}$ at a fixed value of -60423 J/mol.

Supplementary Table 6. Sequences of Oligonucleotides used in thermodynamic model and analysis.

**Standard probe/standard blocker system**	
Probe-1	/5`BHQ1/AGCATAAGCAACACCCTT/3`6-FAM/
MT-1	AAGGGTGTTGCTTATGCT
WT-1	AAGGGAGTTGCTTATGCT
Blocker-1	ATAAGCAACTCCCTT
**Strand displacement probe/strand displacement blocker system**	
Probe-2-F	/5`6-FAM/GCGAACAGTCAAGATCACAGATTTTGG
Probe-2-Q	GCCCGCCCAAAATCTGTGATCTTGACTGTTCGC/3`BHQ1/
MT-2	GTCAAGATCACAGATTTTGGGCGGGC
WT-2	GTCAAGATCACAGATTTTGGGCTGGC
Blocker-2-S	TGTTAATA GTCAAGATCACAGATTTTGG
Blocker-2-L	GCCAGCCCAAAATCTGTGATCTTGACTATTAACA
**Strand displacement probe/standard blocker system**	
Probe-3-F	TGGTGACGTAGGCAAGAGTGAGCTACA/3`6-FAM/
Probe-3-Q	/5`BHQ1/TGTAGCTCACTCTTGCCTACGTCACCAGCTCCAACT
MT-3	AGTTGGAGCTGGTGACGTAGGCAAGAGTGAGCTACA
WT-3	AGTTGGAGCTGATGACGTAGGCAAGAGTGAGCTACA
Blocker-3	ACGTCATCAGCTCCAACT
**Dissociative 4-Way SELECT system**	
Probe-4-F	AAGGGTGTTGCTTATGCTAGCGTAGG/3`6-FAM/
Probe-4-Q	/5`BHQ1/CCTACGCTAGCATAAGCAACACCCTTCCACTGTACTTCATACATG
MT-4	GTACAGTGGAAGGGTGTTGCTTATGCT
WT-4	GTACAGTGGAAGGGGGTTGCTTATGCT
Blocker-4	AGCATAAGCAACCCCCTT
**Non-dissociative 4-Way SELECT system**	
Probe-5-F	/5`6-FAM/TTGGAGCTAGTGGCACGAATCGAAT
Probe-5-Q	ATTCGATTCGTGCCACTAGCTCCAAC/Int Dabcyl-dT/ACCACAAGTTTATAT
MT-5	ATATAAACTTGTGGTAGTTGGAGCTAGTGGCA
WT-5	ATATAAACTTGTGGTAGTTGGAGTTAGTGGCA
Blocker-5	TGCCACTAACTCCAA

Supplementary Table 7. Thermodynamic parameters used in thermodynamic model and analysis.[a]

Systems	ΔG_{BM}	ΔG_{BW}	ΔG_{PM}	ΔG_{PW}	ΔG_{PBM}	ΔG_{PBW}	ΔG_{BP}
Standard probe/standard blocker system	-42.30	-54.60	-47.61	-32.99	-	-	-
Strand displacement probe/strand displacement blocker system	0.58	-11.52	6.61	27.76	-	-	-
Strand displacement probe/standard blocker system	-91.77	-101.40	-55.19	-34.87	-	-	-32.17
Dissociative 4-Way SELECT system	-61.79	-71.42	-1.5	14.24	-1.5	37.88	-
Non-dissociative 4-Way SELECT system	-44.53	-60.42	0	10.32	0	32.36	-

[a]The unit of the free energy changes is kJ/mol

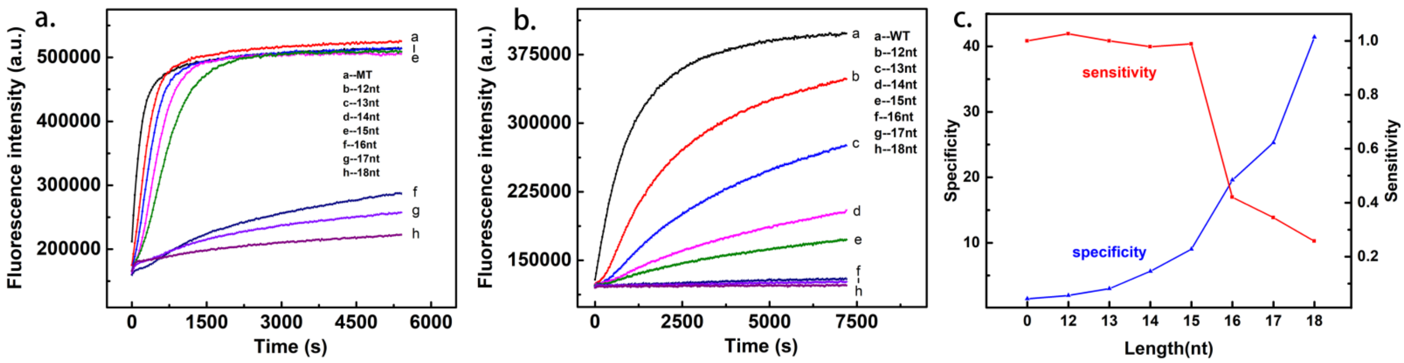

Supplementary Figure 5. The influence of the length of blocker on Strand displacement probe/standard blocker system. (a-b) The fluorescence intensity of strand displacement probe/standard blocker system toward MT (a) and WT (b) with the length of blocker ranging from 12nt to 18nt. The concentration of blocker was fixed at 2.4uM. (c) Experimental results of the sensitivity and specificity of the Strand displacement probe/standard blocker system over the length of blocker.

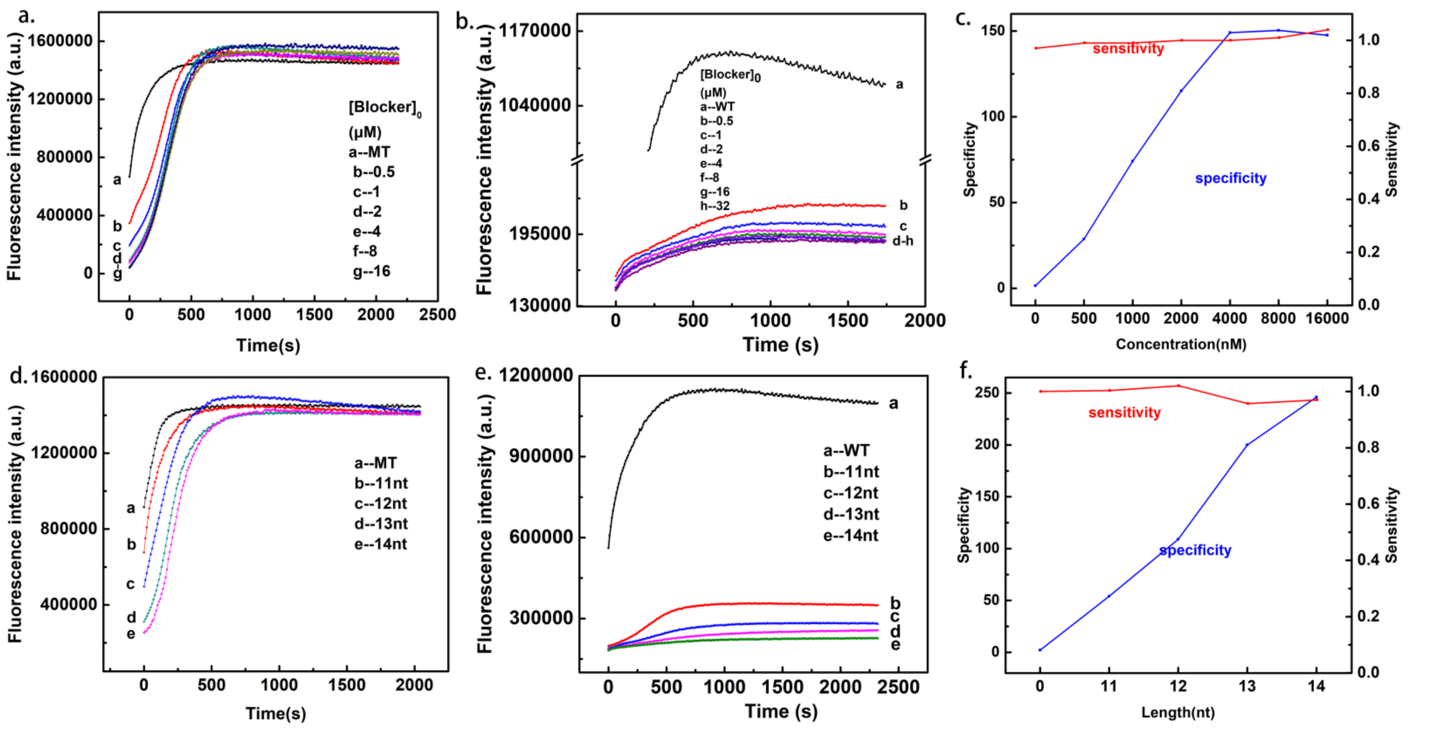

Supplementary Figure 6. The influence of the concentration and length of blocker on the non-dissociative 4-Way SELECT system. (a-b) The fluorescence intensity of the non-dissociative 4-Way SELECT system toward MT (a) and WT (b) with the concentration of blocker ranging from 0uM to 4uM. The length of blocker was fixed at 18nt. (c) Experimental results of the sensitivity and specificity of the non-dissociative 4-Way SELECT system over [Blocker]$_0$.(d-e) The fluorescence intensity of the non-dissociative 4-Way SELECT system toward MT (d) and WT (e) with the length of blocker ranging from 11nt to 14nt. The concentration of blocker was fixed at 4uM. (f) Experimental results of the sensitivity and specificity of the non-dissociative 4-Way SELECT system over [Blocker]$_0$.

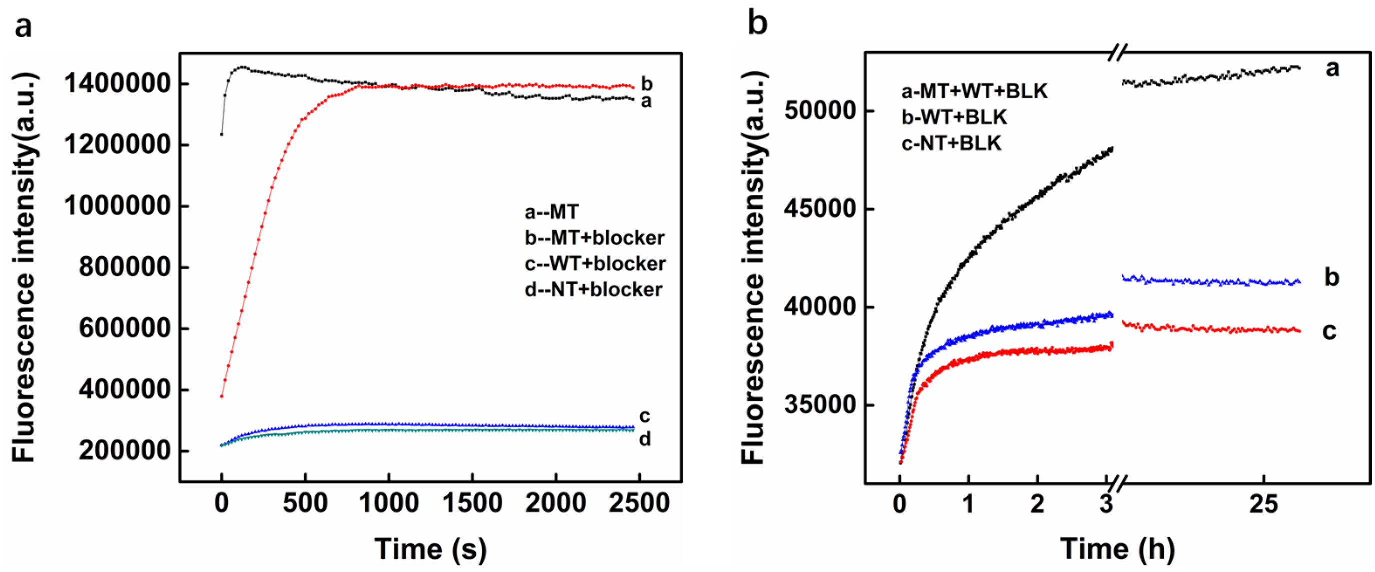

Supplementary Figure 7. The discrimination ability of the dissociative 4-Way SELECT system toward G:G mismatch.(a) The fluorescence intensity of the system toward MT, MT + Blocker, WT + Blocker, and NT + Blocker. (b) The fluorescence intensity of the system toward 10nM MT+1000nM WT+8000nM Blocker,1000nM WT+8000nM Blocker,1000nM NT+8000nM Blocker.

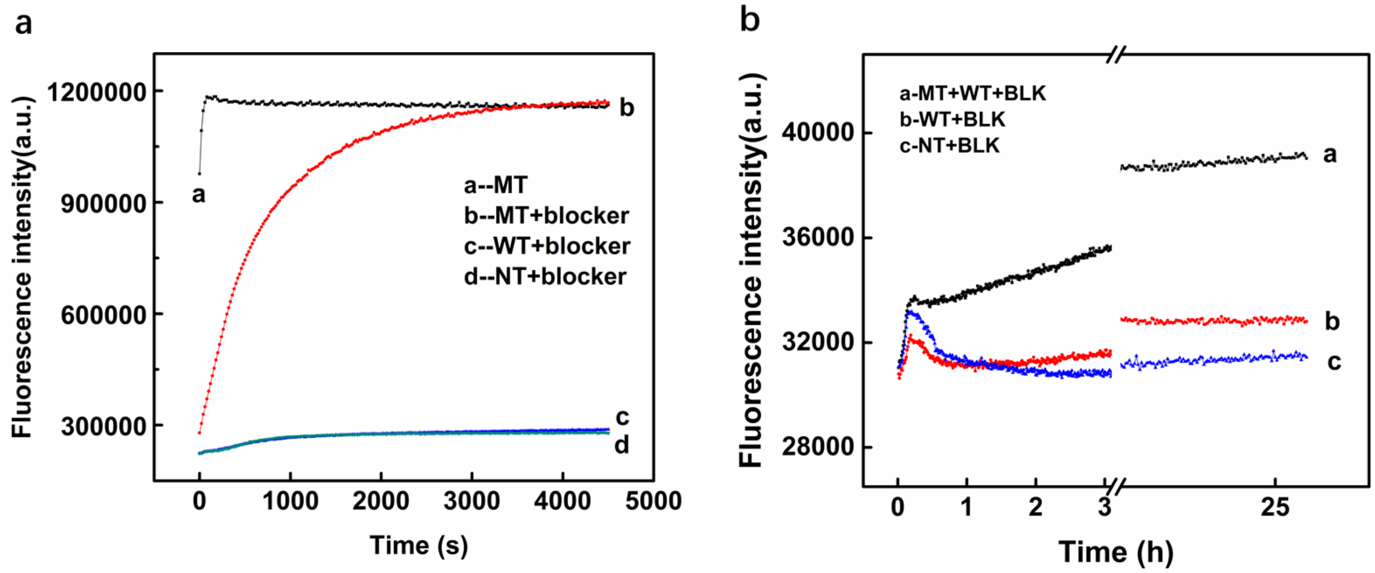

Supplementary Figure 8. The discrimination ability of the dissociative 4-Way SELECT system toward A:G mismatch. (a) The fluorescence intensity of the system toward MT, MT + Blocker, WT + Blocker, and NT + Blocker. (b) The fluorescence intensity of the system toward 10nM MT+1000nM WT+8000nM Blocker, 1000nM WT+8000nM Blocker, 1000nM NT+8000nM Blocker.

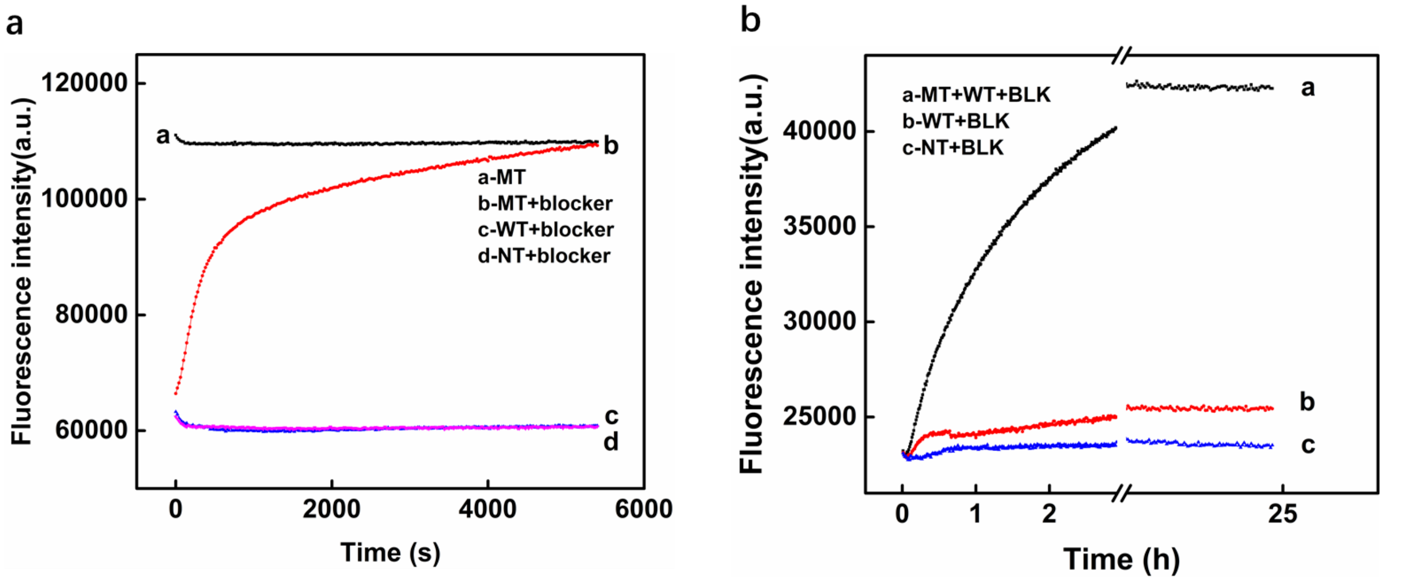

Supplementary Figure 9. The discrimination ability of the dissociative 4-Way SELECT system toward A:A mismatch. (a) The fluorescence intensity of the system toward MT, MT + Blocker, WT + Blocker, and NT + Blocker. (b) The fluorescence intensity of the system toward 10nM MT+1000nM WT+8000nM Blocker,1000nM WT+8000nM Blocker,1000nM NT+8000nM Blocker.

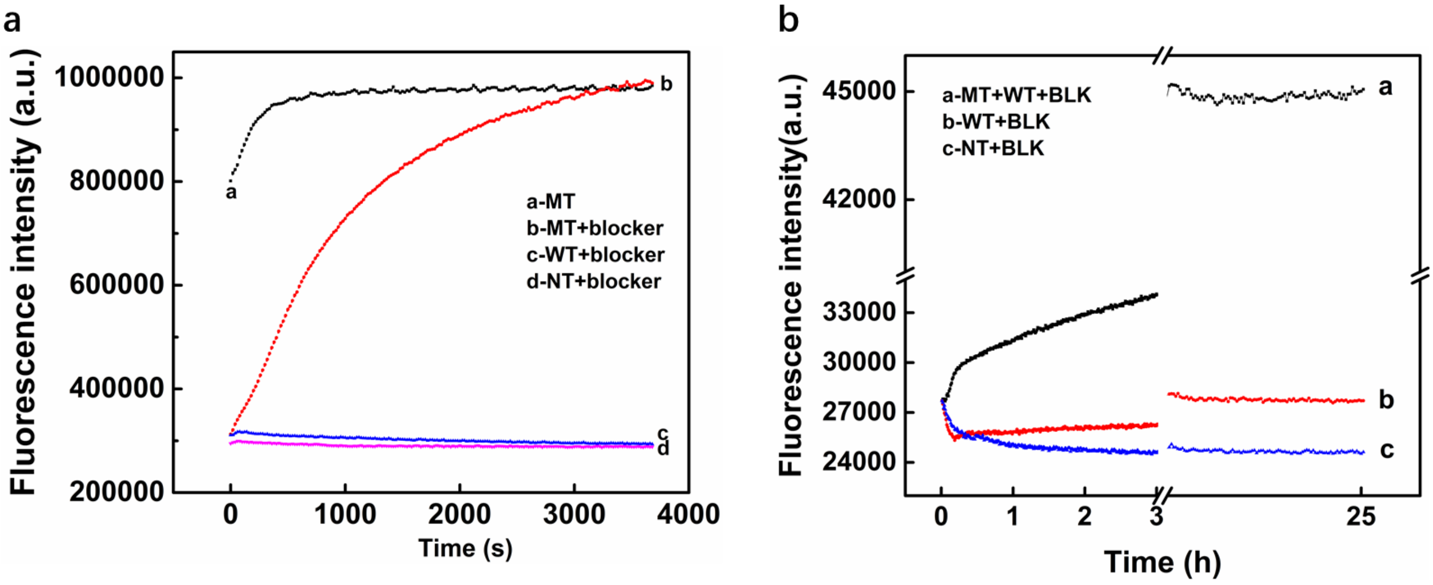

Supplementary Figure 10. The discrimination ability of the dissociative 4-Way SELECT system toward G:T mismatch. (a) The fluorescence intensity of the system toward MT, MT + Blocker, WT + Blocker, and NT + Blocker. (b) The fluorescence intensity of the system toward 10nM MT+1000nM WT+8000nM Blocker,1000nM WT+8000nM Blocker,1000nM NT+8000nM Blocker.

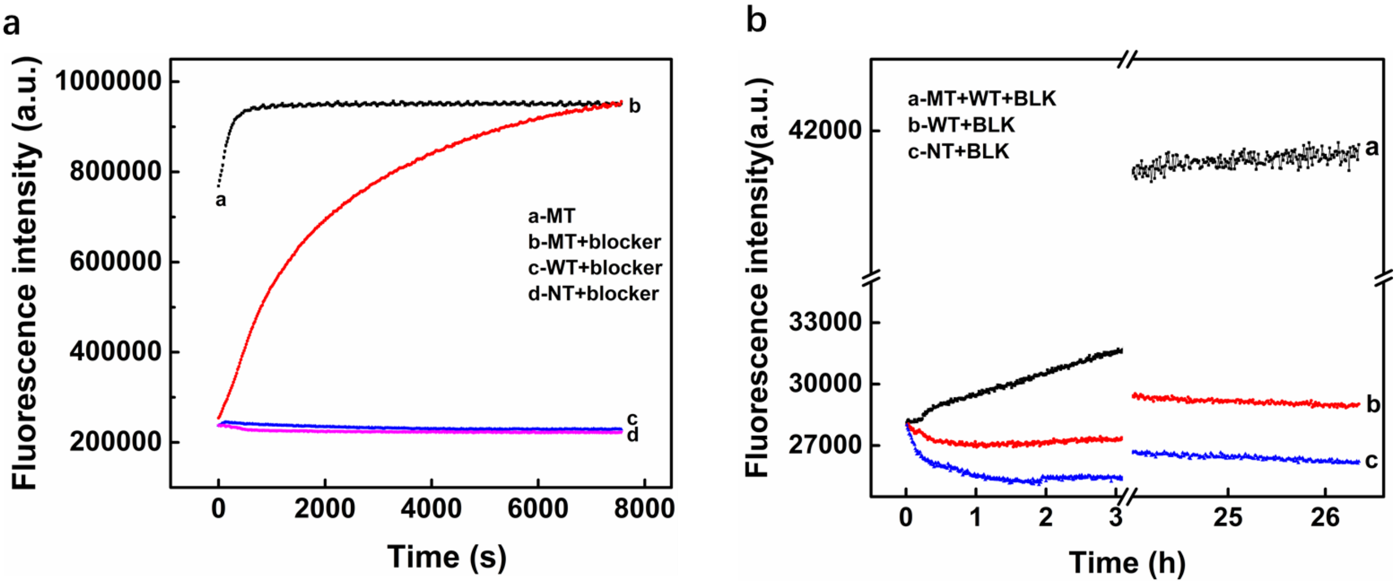

Supplementary Figure 11. The fluorescence intensity of the dissociative 4-Way SELECT system toward miRNA-424, T:T mismatch. (a) The fluorescence intensity of the system toward MT, MT + Blocker, WT + Blocker, and NT + Blocker. (b) The fluorescence intensity of the system toward 10nM MT+1000nM WT+8000nM Blocker,1000nM WT+8000nM Blocker,1000nM NT+8000nM Blocker.

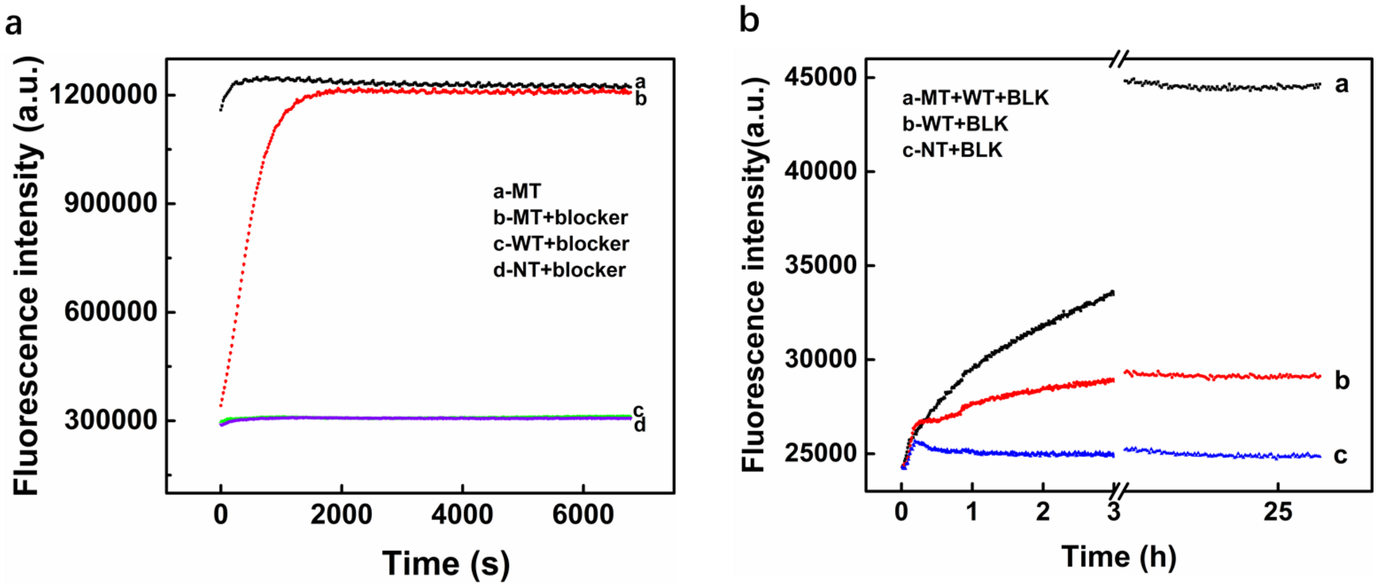

Supplementary Figure 12. The fluorescence intensity of the dissociative 4-Way SELECT system toward miRNA-424, T:G mismatch. (a) The fluorescence intensity of the system toward MT, MT + Blocker, WT + Blocker, and NT + Blocker. (b) The fluorescence intensity of the system toward 10nM MT+1000nM WT+8000nM Blocker,1000nM WT+8000nM Blocker,1000nM NT+8000nM Blocker.

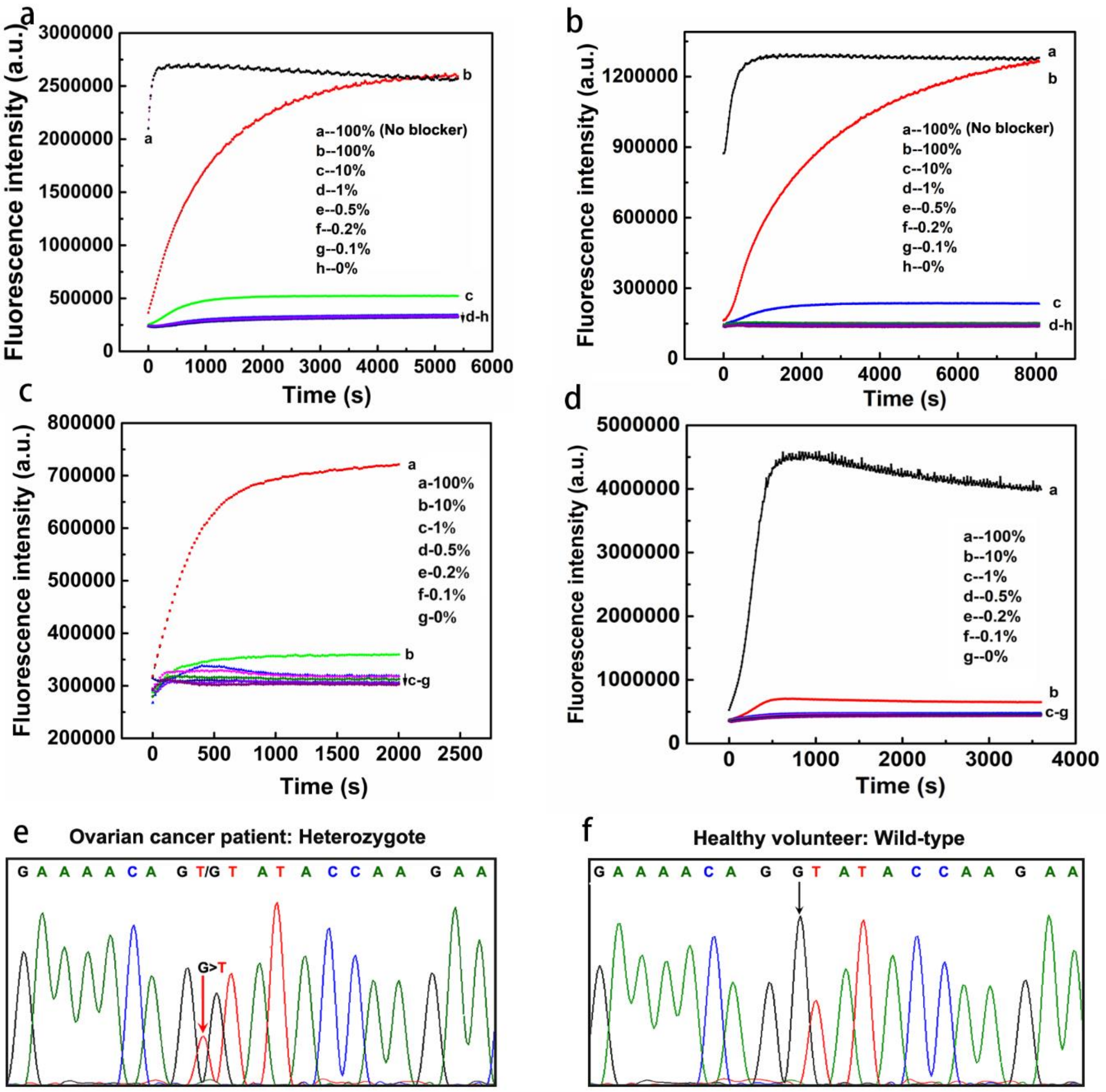

Supplementary Figure 13. Kinetic traces for Post-PCR detection of low-abundance point mutation and the sequencing results of the blood samples. (a-b) Detection of low-abundance synthesized MT by dissociative 4-Way SELECT system. (c-d) Post-PCR detection of low-abundance MT using dissociative (c) and non-dissociative (d) 4-Way SELECT system. (e-f) The sequencing results of the blood samples of an ovarian cancer patient (e) and a healthy volunteer (f).

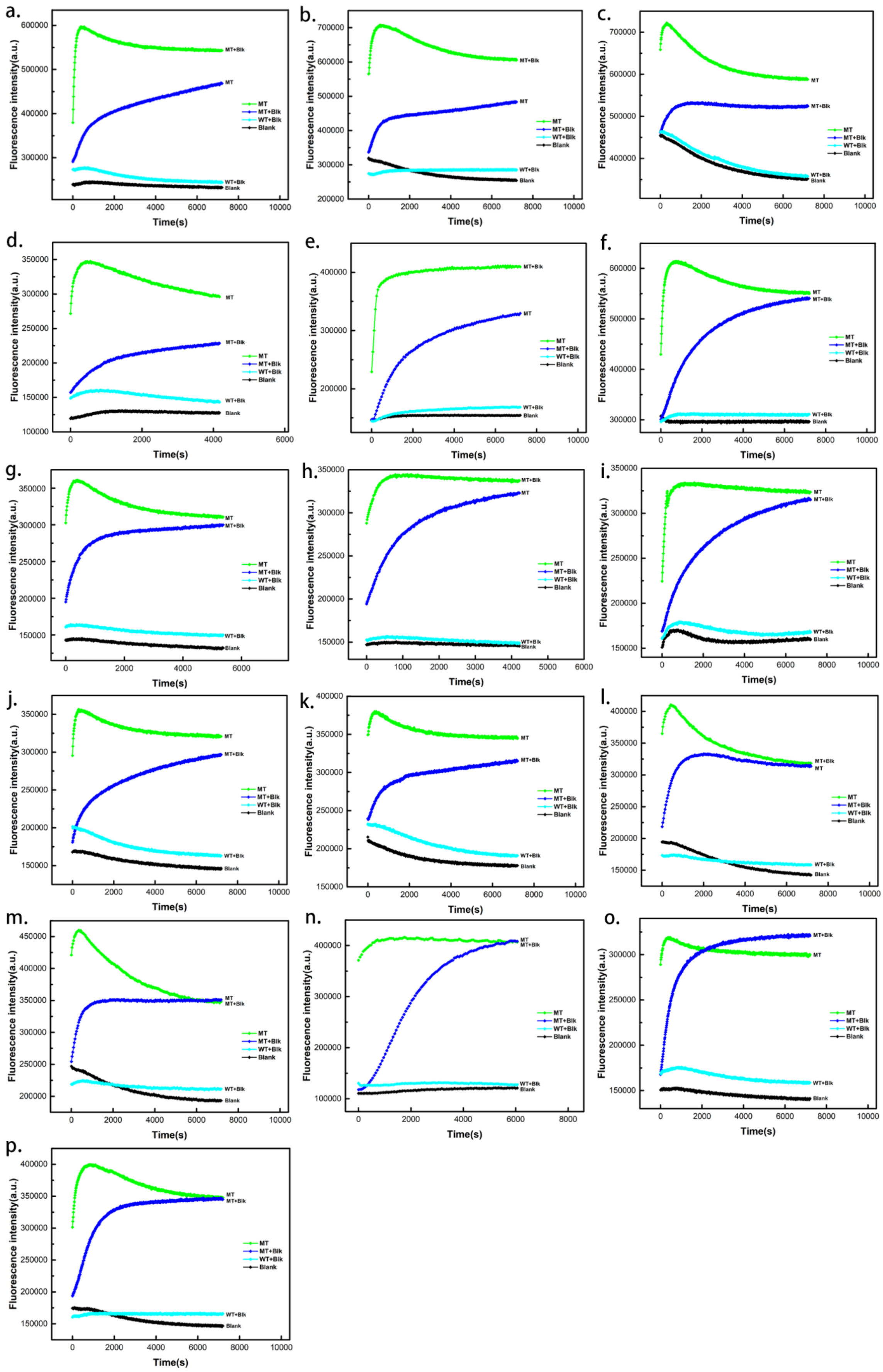

Supplementary Figure 14. Fluorescent kinetic traces for Multiplexed identification of SNVs. (a) 1-MTHFR (rs1801133 C>T), (b) 1-MTRR (rs1801394 A>G), (c) 2-MTHFR (rs1801131 A>C), (d) BRAF-V600E, (e) BRCA1 (rs1006116184 A>C), (f) EGFR-T790E, (g) KRAS-G12A, (h) KRAS-G12C, (i) KRAS-G12D, (j) KRAS-G12S, (k) KRAS-G12V, (l) KRAS-G13C, (m) KRAS-G13D, (n) KRAS-G13S, (o) KRAS-G13V, (p) KRAS-Q61H.

Supplementary Table 8. DNA sequences used in the strand displacement probe/standard blocker system.

Probe-3-F	TGGTGACGTAGGCAAGAGTGAGCTACA/3`6-FAM/
Probe-3-Q	/5`BHQ1/TGTAGCTCACTCTTGCCTACGTCACCAGCTCCAACT
MT	AGTTGGAGCTGGTGACGTAGGCAAGAGTGAGCTACA
WT	AGTTGGAGCTGATGACGTAGGCAAGAGTGAGCTACA
Blocker-12	ACGTCATCAGCT
Blocker-13	ACGTCATCAGCTC
Blocker-14	ACGTCATCAGCTCC
Blocker-15	ACGTCATCAGCTCCA
Blocker-16	ACGTCATCAGCTCCAA
Blocker-17	ACGTCATCAGCTCCAAC
Blocker-18	ACGTCATCAGCTCCAACT

Supplementary Table 9. DNA sequences used in the investigation of the influence of $[Blocker]_0$ and $-\Delta G_{BW}$ on the sensitivity and specificity of dissociative 4-Way SELECT system.

Probe-4-F	AAGGGTGTTGCTTATGCTAGCGTAGG/**3`6-FAM/**
Probe-4-Q	/5`BHQ1/CCTACGCTAGCATAAGCAACACCCTTCCACTGTACTTCATACATG
EGFR-AG-MT-9-7	GTACAGTGGAAGGGTGTTGCTTATGCT
EGFR-AG-WT-9-7	GTACAGTGGAAGGGGGTTGCTTATGCT
EGFR-AG-NT-9-7	GTACAGTGGAAGGG
EGFR-AG-Blocker-11	GCAACCCCCTT
EGFR-AG-Blocker-12	AGCAACCCCCTT
EGFR-AG-Blocker-13	AAGCAACCCCCTT
EGFR-AG-Blocker-14	TAAGCAACCCCCTT
EGFR-AG-Blocker-15	ATAAGCAACCCCCTT
EGFR-AG-Blocker-16	CATAAGCAACCCCCTT
EGFR-AG-Blocker-17	GCATAAGCAACCCCCTT
EGFR-AG-Blocker-18	AGCATAAGCAACCCCCTT

Supplementary Table 10. DNA sequences used in the investigation of the influence of $[Blocker]_0$ and $-\Delta G_{BW}$ on the sensitivity and specificity of non-dissociative 4-Way SELECT system.

Probe-5-F	/5`6-FAM/TTGGAGCTAGTGGCACGAATCGAAT
Probe-5-Q	ATTCGATTCGTGCCACTAGCTCCAAC/IntDabcyldT/ACCACAAGTTTATAT
KRAS-G12S-MT	ATATAAACTTGTGGTAGTTGGAGCTAGTGGC
KRAS-G12S-WT	ATATAAACTTGTGGTAGTTGGAGCTGGTGGC
KRAS-G12S-NT	ATATAAACTTGTGGTAGTTGGAGCT
KRAS-G12S-Blocker-11	ACCAGCTCCAA
KRAS-G12S-Blocker-12	CACCAGCTCCAA
KRAS-G12S-Blocker-13	CCACCAGCTCCAA
KRAS-G12S-Blocker-14	GCCACCAGCTCCAA

Supplementary Table 11. DNA sequences used in determination of the specificity and IF of the dissociative 4-Way SELECT system toward 6 types of mismatches.

**EGFR-A:G mismatch**	
Probe-4-F	AAGGGTGTTGCTTATGCTAGCGTAGG/3`6-FAM/
Probe-4-Q	/5`BHQ1/CCTACGCTAGCATAAGCAACACCCTTCCACTGTACTTCATACATG
EGFR-AG-MT-11-7	AAGTACAGTGGAAGGGTGTTGCTTATGCT
EGFR-AG-WT-11-7	AAGTACAGTGGAAGGGGGTTGCTTATGCT
EGFR-AG-NT-11-7	AAGTACAGTGGAAGGG
EGFR-AG-Blocker-18	AGCATAAGCAACCCCCTT
**EGFR-G:G mismatch**	
Probe-4-F	AAGGGTGTTGCTTATGCTAGCGTAGG/3`6-FAM/
Probe-4-Q	/5`BHQ1/CCTACGCTAGCATAAGCAACACCCTTCCACTGTACTTCATACATG

EGFR-GG-MT-11-7	AAGTACAGTGGAAGGGTGTTGCTTATGCT
EGFR-GG-WT-11-7	AAGTACAGTGGAAGGGTGTTGGTTATGCT
EGFR-GG-NT-11-7	CTACGGTTCAACACCCTT
EGFR-GG-Blocker-18	AGCATAACCAACACCCTT
**MiRNA-424-T:T mismatch**	
Probe-6-F	TAGGAATACGCGTTCGGTAGATTTCTTATGC/3`6-FAM/
Probe-6-Q	/5`BHQ1/GCATAAGAAATCTACCGAACGCGTATTCCTACCACCAAATACCCA C
MiRNA-424-TT-MT-11-9	GTATTTGGTGGTAGGAATACGCGTTCGGTAGAT
MiRNA-424-TT-WT-11-9	GTATTTGGTGGTAGGATTACGCGTTCGGTAGAT
MiRNA-424-TT-NT-11-9	GTATTTGGTGGTAGGA
MiRNA-424-TT-Blocker-19	TACCGAACGCGTAATCCTA
**MiRNA-424-G:T mismatch**	
Probe-6-F	TAGGAATACGCGTTCGGTAGATTTCTTATGC/3`6-FAM/
Probe-6-Q	/5`BHQ1/GCATAAGAAATCTACCGAACGCGTATTCCTACCACCAAATACCCA C
MiRNA-424-GT-MT-11-9	GTATTTGGTGGTAGGAATACGCGTTCGGTAGAT
MiRNA-424-GT-WT-11-9	GTATTTGGTGGTAGGAATATGCGTTCGGTAGAT
MiRNA-424-GT-NT-11-9	GTATTTGGTGGTAGGAATA
MiRNA-424-GT-Blocker-19	TACCGAACGCGTAATCCTA
**KRAS-G13D-T:G mismatch**	
Probe-7-F	TGGTGACGTAGGCAAGAGTGAGCTACA/3`6-FAM/
Probe-7-Q	/5`BHQ1/TGTAGCTCACTCTTGCCTACGTCACCAGCTCCAACTACCACA
KRAS-G13D-TG-MT-11-7	GTAGTTGGAGCTGGTGACGTAGGCAAGAGTG
KRAS-G13D-TG-WT-11-7	GTAGTTGGAGCTGGTGGCGTAGGCAAGAGTG
KRAS-G13D-TG-NT-11-7	GTAGTTGGAGCTGGTG
KRAS-G13D-TG-Blocker-17	CTCTTGCCTACGCCACC
**KRAS-A:A mismatch**	
Probe-7-F	TGGTGACGTAGGCAAGAGTGAGCTACA/3`6-FAM/
Probe-7-Q	/5`BHQ1/TGTAGCTCACTCTTGCCTACGTCACCAGCTCCAACTACCACA
KRAS-AA-MT-11-7	GTAGTTGGAGCTGGTGACGTAGGCAAGAGTG
KRAS-AA-WT-11-7	GTAGTTGGAGCTGGTGACGAAGGCAAGAGTG
KRAS-AA-NT-11-7	GTAGTTGGAGCTGGTGACG
KRAS-AA-Blocker-17	CTCTTGCCTTCGTCACC

Supplementary Table 12. DNA sequences used in Post-PCR detection of low-abundance point mutation.

BRCA80358053-Probe-F	AACAGTTATACCTAGAACAGGCTATAAAACTCCGTCGGCGAGAC
BRCA80358053-Probe-Q	ACACGTGTGACGGCCAAATATAGCCTGTTCTTGGTATAACTGTTTTCAT AACAACATG
BRCA80358053-FP	ATGCTCGTGTACAAGTTTGCC
BRCA80358053-RP	TTATGCAGCAGATGCAAGGT
BRCA80358053-Blocker-18	GTTCTTGGTATACCTGTT
BRCA80358053-Blocker-25	GTTCTTGGTATACCTGTTATTCTCT
KRAS-G12S-MT-108nt	GCCTGCTGAAAATGACTAAGAATATAAACTTGTGGTAGTTGGAGCTAG TGGCGTAGGCAAGAGTGCCTTGACGATACAGCTAATTCAGAATCATTT TGTGGACGAATA
KRAS-G12S-WT-108nt	GCCTGCTGAAAATGACTAAGAATATAAACTTGTGGTAGTTGGAGCTGG TGGCGTAGGCAAGAGTGCCTTGACGATACAGCTAATTCAGAATCATTT TGTGGACGAATA
KRAS-G13D-TG-MT-11-7-108nt	GCCTGCTGAAAATGACTAAGAATATAAACTTGTGGTAGTTGGAGCTGG TGACGTAGGCAAGAGTGCCTTGACGATACAGCTAATTCAGAATCATTT TGTGGACGAATA
KRAS-G13D-TG-WT-11-7-108nt	GCCTGCTGAAAATGACTAAGAATATAAACTTGTGGTAGTTGGAGCTGG TGGCGTAGGCAAGAGTGCCTTGACGATACAGCTAATTCAGAATCATTT

	TGTGGACGAATA

Supplementary Table 13. DNA sequences used in multiplexed identification of SNVs.

Shared-FAM	GTCTCGCCGACGGAG/**3`6-FAM/**
Shared-BHQ	/5`BHQ1/GGCCGTCACACGTGT
**KRAS-G12A**	
KRAS-G12A-Probe-S	AGCTGCTGGCGTAGGCAAGAGTGCCTAAACTCCGTCGGCGAGAC
KRAS-G12A-Probe-L	ACACGTGTGACGGCCAAAAGGCACTCTTGCCTACGCCAGCAGCTCCAACTACCCAAGT
KRAS-G12A-Blocker-18	TTGCCTACGCCACCAGCT
KRAS-G12A-MT	TGGGTAGTTGGAGCTGCTGGCGTAGGCAA
KRAS-G12A-WT	TGGGTAGTTGGAGCTGGTGGCGTAGGCAA
**KRAS-G12C**	
KRAS-G12C-Probe-S	AGCTTGTGGCGTAGGCAAGAGTGCCTAAACTCCGTCGGCGAGAC
KRAS-G12C-Probe-L	ACACGTGTGACGGCCAAAAGGCACTCTTGCCTACGCCACAAGCTCCAACTACCCAAGT
KRAS-G12C-Blocker-18	TTGCCTACGCCACCAGCT
KRAS-G12C-MT	TGGGTAGTTGGAGCTTGTGGCGTAGGCAA
KRAS-G12C-WT	TGGGTAGTTGGAGCTGGTGGCGTAGGCAA
**KRAS-G12D**	
KRAS-G12D-Probe-S	AGCTGATGGCGTAGGCAAGAGTGCCTAAACTCCGTCGGCGAGAC
KRAS-G12D-Probe-L	ACACGTGTGACGGCCAAAAGGCACTCTTGCCTACGCCATCAGCTCCAACTACCCAAGT
KRAS-G12D-Blocker-18	TTGCCTACGCCACCAGCT
KRAS-G12D-MT	TGGGTAGTTGGAGCTGATGGCGTAGGCAA
KRAS-G12D-WT	TGGGTAGTTGGAGCTGGTGGCGTAGGCAA
**KRAS-G12S**	
KRAS-G12S-Probe-S	AGCTAGTGGCGTAGGCAAGAGTGCCTAAACTCCGTCGGCGAGAC
KRAS-G12S-Probe-L	ACACGTGTGACGGCCAAAAGGCACTCTTGCCTACGCCACTAGCTCCAACTACCCAAGT
KRAS-G12S-Blocker-18	TTGCCTACGCCACCAGCT
KRAS-G12S-MT	TGGGTAGTTGGAGCTAGTGGCGTAGGCAA
KRAS-G12S-WT	TGGGTAGTTGGAGCTGGTGGCGTAGGCAA
**KRAS-G12V**	
KRAS-G12V-Probe-S	AGCTGTTGGCGTAGGCAAGAGTGCCTAAACTCCGTCGGCGAGAC
KRAS-G12V-Probe-L	ACACGTGTGACGGCCAAAAGGCACTCTTGCCTACGCCAACAGCTCCAACTACCCAAGT
KRAS-G12V-Blocker-18	TTGCCTACGCCACCAGCT
KRAS-G12V-MT	TGGGTAGTTGGAGCTGTTGGCGTAGGCAA
KRAS-G12V-WT	TGGGTAGTTGGAGCTGGTGGCGTAGGCAA
**KRAS-G13C**	
KRAS-G13C-Probe-S	AGCTGGTTGCGTAGGCAAGAGTGCCTAAACTCCGTCGGCGAGAC
KRAS-G13C-Probe-L	ACACGTGTGACGGCCAAAAGGCACTCTTGCCTACGCAACCAGCTCCAACTACCCAAGT
KRAS-G13C-Blocker-18	TTGCCTACGCCACCAGCT
KRAS-G13C-MT	TGGGTAGTTGGAGCTGGTTGCGTAGGCAA
KRAS-G13C-WT	TGGGTAGTTGGAGCTGGTGGCGTAGGCAA
**KRAS-G13D**	
KRAS-G13D-Probe-S	AGCTGGTGACGTAGGCAAGAGTGCCTAAACTCCGTCGGCGAGAC
KRAS-G13D-Probe-L	ACACGTGTGACGGCCAAAAGGCACTCTTGCCTACGTCACCAGCTCCAACTACCCAAGT
KRAS-G13D-Blocker-18	TTGCCTACGCCACCAGCT
KRAS-G13D-MT	TGGGTAGTTGGAGCTGGTGACGTAGGCAA
KRAS-G13D-WT	TGGGTAGTTGGAGCTGGTGGCGTAGGCAA
**KRAS-G13S**	
KRAS-G13S-Probe-S	AGCTGGTAGCGTAGGCAAGAGTGCCTAAACTCCGTCGGCGAGAC
KRAS-G13S-Probe-L	ACACGTGTGACGGCCAAAAGGCACTCTTGCCTACGCTACCAGCTCCA

	ACTACCACAAG
KRAS-G13S-Blocker-18	TTGCCTACGCCACCAGCT
KRAS-G13S-MT	TGGGTAGTTGGAGCTGGTAGCGTAGGCAA
KRAS-G13S-WT	TGGGTAGTTGGAGCTGGTGGCGTAGGCAA
**KRAS-G13V**	
KRAS-G13V-Probe-S	AGCTGGTGTCGTAGGCAAGAGTGCCTAAACTCCGTCGGCGAGAC
KRAS-G13V-Probe-L	ACACGTGTGACGGCCAAAAGGCACTCTTGCCTACGACACCAGCTCCA ACTACCCAAGT
KRAS-G13V-Blocker-18	TTGCCTACGCCACCAGCT
KRAS-G13V-MT	TGGGTAGTTGGAGCTGGTGTCGTAGGCAA
KRAS-G13V-WT	TGGGTAGTTGGAGCTGGTGGCGTAGGCAA
**KRAS-Q61H**	
KRAS-Q61H-Probe-S	GGTCACGAGGAGTACAGTGCAATGAGAAACTCCGTCGGCGAGAC
KRAS-Q61H-Probe-L	ACACGTGTGACGGCCAAACTCATTGCACTGTACTCCTCGTGACCTGCT GTGTCGAGAA
KRAS-Q61H-Blocker-18	ACTGTACTCCTCTTGACC
KRAS-Q61H-MT	TCGACACAGCAGGTCACGAGGAGTACAGT
KRAS-Q61H-WT	TCGACACAGCAGGTCAAGAGGAGTACAGT
**BRAF-D594G**	
BRAF-D594G-Probe-S	AGGTGGTTTTGGTCTAGCTACAGTGAGGGCTCCGTCGGCGAGAC
BRAF-D594G-Probe-L	ACACGTGTGACGGCCGGGTCACTGTAGCTAGACCAAAACCACCTATTT TTACTGTGAG
BRAF-D594G-Blocker-18	GCTAGACCAAAATCACCT
BRAF-D594G-MT	ACAGTAAAAATAGGTGGTTTTGGTCTAGC
BRAF-D594G-WT	ACAGTAAAAATAGGTGATTTTGGTCTAGC
**EGFR-V600E**	
EGFR-V600E-Probe-S	TACAGAGAAATCTCGATGGAGTGGGTTTTCTCCGTCGGCGAGAC
EGFR-V600E-Probe-L	ACACGTGTGACGGCCTTTACCCACTCCATCGAGATTTCTCTGTAGCTAG ACCAAAATC
EGFR-V600E-Blocker-18	CATCGAGATTTCACTGTA
EGFR-V600E-MT	TTTGGTCTAGCTACAGAGAAATCTCGATG
EGFR-V600E-WT	TTTGGTCTAGCTACAGTGAAATCTCGATG
**1-MTHFR (rs1801133 C>T)**	
1-MTHFR-Probe-S	GGGAGTCGATTTCATCATCACGCAGCAAACTCCGTCGGCGAGAC
1-MTHFR-Probe-L	ACACGTGTGACGGCCAAAGCTGCGTGATGATGAAATCGACTCCCGCA GACACCTTCTC
1-MTHFR-Blocker-18	ATGATGAAATCGGCTCCC
1-MTHFR-MT	AAGGTGTCTGCGGGAGTCGATTTCATCAT
1-MTHFR-WT	AAGGTGTCTGCGGGAGCCGATTTCATCAT
**1-MTRR (rs1801394 A>G)**	
1-MTRR-Probe-S	GAAATGTGTGAGCAAGCTGTGGTACAAAACTCCGTCGGCGAGAC
1-MTRR-Probe-L	ACACGTGTGACGGCCAAATGTACCACAGCTTGCTCACACATTTCTTCT GCGATGGCCT
1-MTRR-Blocker-18	AGCTTGCTCACATATTTC
1-MTRR-MT	CCATCGCAGAAGAAATGTGTGAGCAAGCT
1-MTRR-WT	CCATCGCAGAAGAAATATGTGAGCAAGCT
**2-MTHFR (rs1801131 A>C)**	
2-MTHFR-Probe-S	TGAAGCAAGTGTCTTTGAAGTCTTCGAAACTCCGTCGGCGAGAC
2-MTHFR-Probe-L	ACACGTGTGACGGCCAAACGAAGACTTCAAAGACACTTTCTTCACTG GTCAGCTCCTC
2-MTHFR-Blocker-18	TCAAAGACACTTTCTTCA
2-MTHFR-MT	GAGCTGACCAGTGAAGCAAGTGTCTTTGA
2-MTHFR-WT	GAGCTGACCAGTGAAGAAAGTGTCTTTGA
**BRAC1**	
BRAC1-Probe-S	ATGGCCGATTTCCAAGGGGAGTGCCTAAACTCCGTCGGCGAGAC
BRAC1-Probe-L	ACACGTGTGACGGCCAAAAGGCACTCCCCTTGGAAATCGGCCATGAG CACAAAATTAT
BRAC1-Blocker-18	CCCTTGGAAATCTGCCAT

BRAC1-MT	ATTTTGTGCTCATGGCCGATTTCCAAGGG
BRAC1-WT	ATTTTGTGCTCATGGCAGATTTCCAAGGG
