# Supplementary material for: Thermodynamics and kinetics guided probe design for uniformly sensitive and specific DNA hybridization without optimization
Source: Nat Commun. 2019 Oct 14;10:4675. doi: 10.1038/s41467-019-12593-9 (PMC6791858; doi:10.1038/s41467-019-12593-9)
Supplement: Supplementary file 3 — Reporting Summary [file 41467_2019_12593_MOESM3_ESM.pdf]

nature research

Corresponding author(s): Xianjin Xiao
Last updated by author(s): Sep 16, 2019

Reporting Summary

Nature Research wishes to improve the reproducibility of the work that we publish. This form provides structure for consistency and transparency in reporting. For further information on Nature Research policies, see Authors & Referees and the Editorial Policy Checklist .

Statistics

For all statistical analyses, confirm that the following items are present in the figure legend, table legend, main text, or Methods section.

n/a	Confirmed	
☐	☒	The exact sample size (n) for each experimental group/condition, given as a discrete number and unit of measurement
☐	☒	A statement on whether measurements were taken from distinct samples or whether the same sample was measured repeatedly
☒	☐	The statistical test(s) used AND whether they are one- or two-sided<br>*Only common tests should be described solely by name; describe more complex techniques in the Methods section.*
☒	☐	A description of all covariates tested
☐	☒	A description of any assumptions or corrections, such as tests of normality and adjustment for multiple comparisons
☐	☒	A full description of the statistical parameters including central tendency (e.g. means) or other basic estimates (e.g. regression coefficient) AND variation (e.g. standard deviation) or associated estimates of uncertainty (e.g. confidence intervals)
☒	☐	For null hypothesis testing, the test statistic (e.g. F, t, r) with confidence intervals, effect sizes, degrees of freedom and P value noted<br>*Give P values as exact values whenever suitable.*
☒	☐	For Bayesian analysis, information on the choice of priors and Markov chain Monte Carlo settings
☐	☒	For hierarchical and complex designs, identification of the appropriate level for tests and full reporting of outcomes
☒	☐	Estimates of effect sizes (e.g. Cohen's d, Pearson's r), indicating how they were calculated

Our web collection on statistics for biologists contains articles on many of the points above.

Software and code

Policy information about availability of computer code

Data collection	The thermodynamic parameters of associated DNA reactions were obtained by NUPACK (http://www.nupack.org/).
Data analysis	Maple 2019 was used to construct theoretical model.<br>OriginPro 8.0 was used to analyze the data and draw the figures.

For manuscripts utilizing custom algorithms or software that are central to the research but not yet described in published literature, software must be made available to editors/reviewers. We strongly encourage code deposition in a community repository (e.g. GitHub). See the Nature Research guidelines for submitting code & software for further information.

Data

Policy information about availability of data

All manuscripts must include a data availability statement. This statement should provide the following information, where applicable:

- Accession codes, unique identifiers, or web links for publicly available datasets
- A list of figures that have associated raw data
- A description of any restrictions on data availability

All the data supporting the findings of this study are available within the paper and the supplementary files. The source data underlying Figures 2, 3, 4, 5 and Supplementary Figures 5 to Supplementary Figure 14 are provided as a Source Data file. All other relevant data are available from the authors upon reasonable request.

Field-specific reporting

Please select the one below that is the best fit for your research. If you are not sure, read the appropriate sections before making your selection.

Life sciences study design

All studies must disclose on these points even when the disclosure is negative.

Sample size	We used genomic DNA samples from a healthy volunteer and an ovarian cancer patient for identification of the SNV in BRCA 1/2 genes. The experiment were conducted in triplets and the results were compared with Sanger sequencing results. We believe it was sufficient to demonstrate the feasibility of our system on clinical samples.
Data exclusions	No data were excluded from analysis.
Replication	All the experimental findings were reliably reproduced.
Randomization	No randomization was used.
Blinding	The investigators were blinded to the information of the two blood samples.

Reporting for specific materials, systems and methods

We require information from authors about some types of materials, experimental systems and methods used in many studies. Here, indicate whether each material, system or method listed is relevant to your study. If you are not sure if a list item applies to your research, read the appropriate section before selecting a response.

Materials & experimental systems

n/a	Involved in the study	
☒	☐	Antibodies
☒	☐	Eukaryotic cell lines
☒	☐	Palaeontology
☒	☐	Animals and other organisms
☐	☒	Human research participants
☒	☐	Clinical data

Methods

n/a	Involved in the study	
☒	☐	ChIP-seq
☒	☐	Flow cytometry
☒	☐	MRI-based neuroimaging

Human research participants

Policy information about studies involving human research participants

Population characteristics	The healthy volunteer: female, 35 years old, wild-type (BRCA rs80358053).<br>The ovarian cancer patient: female, 54 years old, heterozygote (BRCA rs80358053), ovarian cancer stage III.
Recruitment	For the healthy volunteer: female, wild-type of BRCA rs80358053 is recruited.<br>For the ovarian cancer patient: diagnosed with ovarian cancer, homozygote or heterozygote of BRCA rs80358053 is recruited.<br>All participants were recruited from hospital and all participants provided written informed consent for genetic analysis.
Ethics oversight	The study was approved by the Ethics Committee of Tongji Medical College of Huazhong University of Science and Technology

Note that full information on the approval of the study protocol must also be provided in the manuscript.
